# Rottlerin triggers dual degradation of SLC7A11 and GPX4 to drive ferroptosis and chemosensitization in hepatocellular carcinoma

**DOI:** 10.1038/s41420-026-02942-1

**Published:** 2026-01-30

**Authors:** Hongliang Luo, Xiaorui Jin, Chengchang Gao, Qinqin Deng, Linfen Han, Fumin Hu, Rui Tong, Donglin Li, Haoying Yang, Xueli Bian

**Affiliations:** 1 Department of General Surgery, The Second Affiliated Hospital, Jiangxi Medical College, Nanchang University, Nanchang, China; 2 The MOE Basic Research and Innovation Center for the Targeted Therapeutics of Solid Tumors, Jiangxi Provincial Key Laboratory of Tumor Biology, School of Basic Medical Sciences, Nanchang University, Nanchang, China

**Keywords:** Cancer metabolism, Drug development

## Abstract

Natural products have emerged as promising therapeutic agents for targeting redox vulnerabilities in cancer. Rottlerin, a bioactive polyphenol derived from *Mallotus philippinensis*, exhibits broad anticancer properties through autophagy and apoptosis induction. However, its capacity to modulate ferroptosis, a druggable form of iron-dependent cell death, remains unexplored in hepatocellular carcinoma (HCC). Here, we demonstrate that rottlerin potently inhibits HCC proliferation by triggering ferroptosis execution, as evidenced by lipid peroxidation accumulation and ferroptosis inhibitor (ferrostatin-1)-rescued cell death. Strikingly, subtherapeutic doses of rottlerin enhanced the efficacy of clinical ferroptosis inducers (RSL3 and sorafenib), and this chemosensitization effect persisted in PKCδ-depleted models, indicating a target-agnostic mechanism. Mechanistically, rottlerin orchestrates ubiquitin-proteasomal degradation of two central ferroptosis defense nodes: the cystine transporter SLC7A11 and glutathione peroxidase 4 (GPX4), thereby compromising cellular antioxidant capacity. This dual-degradation strategy distinguishes rottlerin from single-target phytochemicals and underlies its robust ferroptosis induction. Our work provides the first demonstration of rottlerin’s ferroptotic activity in HCC, positioning it as a dual degrader capable of overcoming compensatory antioxidant adaptations. These findings advocate for rottlerin’s clinical development either as monotherapy or in rational combinations to augment ferroptosis-targeted HCC treatment.

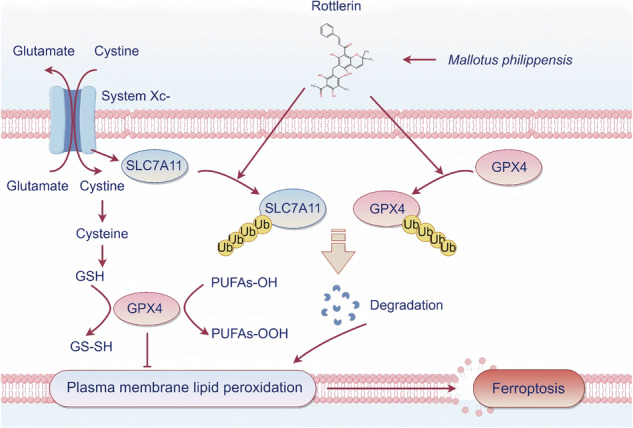

## Introduction

Hepatocellular carcinoma (HCC), the predominant histological subtype of primary liver cancer [1, 2], represents a major global health burden. Recent epidemiological data from the 2022 Global Cancer Statistics reveal that HCC constitutes 4.3% of total cancer diagnoses and accounts for 7.8% of cancer-related mortality worldwide [3]. In China, this malignancy ranks as the fifth most common neoplasm and the second leading cause of cancer death, underscoring its disproportionate impact in Asian populations [4]. HCC has an insidious onset and often lacks specific symptoms in the early stages, making it difficult to detect through routine physical examinations. Consequently, clinical diagnosis typically occurs in the late stages, and the treatment options are limited. This trend of early asymptomatic presentation and late-stage diagnosis underscores the urgent need for more effective HCC treatment strategies to enhance prognosis and life quality in patients with HCC.

Ferroptosis, a type of cell death characterized by iron-dependent lipid peroxidation, plays a significant therapeutic role in HCC. It is characterized by glutathione (GSH) exhaustion, glutathione peroxidase 4 (GPX4) inactivation, excessive free iron, and lipid peroxidation damage [5]. Disorders in iron metabolism can mediate lipid peroxidation processes, leading to the production and accumulation of lipid peroxides within cells [6]. This accumulation can damage the cell’s membrane system, destroy the lipid bilayer, and ultimately cause cell membrane rupture and cell death through ferroptosis [7, 8]. Solute carrier family 7 member 11 (SLC7A11) is the cystine/glutamate antiporter subunit responsible for transporting extracellular cystine into cells [9]. Cystine is a crucial precursor for the synthesis of glutathione (GSH), a potent antioxidant that protects cells from oxidative stress [10]. GPX4 is a key enzyme that uses GSH as a cofactor to reduce peroxidized polyunsaturated fatty acids (PUFAs−OOH), thereby preventing cell membrane lipid peroxidation and which is essential for maintaining cell membrane integrity and preventing ferroptosis [11]. Inhibition of GPX4 enzymatic activity, or its post-translational modifications such as ubiquitination, affects its activity and stability, ultimately altering cellular sensitivity to ferroptosis [12]. SLC7A11 is one of the key regulatory molecules of ferroptosis. Inhibition of SLC7A11 induces ferroptosis by depriving cellular cysteine, which results in GSH depletion, suppression of GPX4 activity, and accumulation of lipid peroxides [13]. Alterations in GPX4 and SLC7A11 protein expression have been reported as predictive markers for the assessment of ferroptosis sensitivity or resistance [14]. The SLC7A11−GSH−GPX4 axis is the primary defense system of cells against ferroptosis [15]. Therefore, targeting the SLC7A11−GSH−GPX4 axis has emerged as a promising strategy to treat cancer by inducing ferroptosis in tumor cells. In recent years, the development of ferroptosis inducers or sensitizers for cancer treatment has become a hot topic.

PKCδ (protein kinase Cδ) is a member of the protein kinase C (PKC) family and functions as a serine/threonine kinase, which plays a crucial role in cellular signal transduction and regulates various processes, including cell proliferation, apoptosis, differentiation, and migration [16–18]. In breast cancer, PKCδ promotes the growth of MDA-MB-231 cells by inhibiting the ERK1/2 pathway [19]. Inhibiting PKCδ can slow cell proliferation and tumor growth in liver cancer cells [20]. In HCC, PKCδ regulates gene expression associated with migration and induces cell cycle arrest at G1 phase in HCC, suggesting that targeted therapy against PKCδ could help prevent the progression of this disease [21].

Rottlerin is a compound derived from *Mallotus philippinensis*. It has been reported to be a specific inhibitor of PKCδ [22]. As a natural polyphenolic compound, rottlerin has demonstrated various properties, including anti-tumor [23], anti-inflammatory [24, 25], antiviral [26, 27], and neuroprotective effects [28]. Additionally, it has been found to inhibit lipid accumulation in adipocytes, exhibiting anti-obesity properties [29]. Rottlerin induces autophagy, apoptosis, and cell cycle arrest in various cancer cells, including HCC, inhibiting tumor cell growth and invasion [30–34], which makes it a promising candidate for application in chemotherapy. However, it remains unclear whether rottlerin can affect the regulation of ferroptosis in HCC cells.

In this study, we systematically investigate the ferroptosis-inducing capacity of rottlerin in HCC models. Our findings reveal a novel mechanism whereby rottlerin promotes proteasomal degradation of SLC7A11 and GPX4, thereby disabling cellular antioxidant defenses and potentiating lipid peroxidation.

## Results

### Rottlerin suppresses HCC cell proliferation in a concentration-dependent manner

**Fig. 1:**
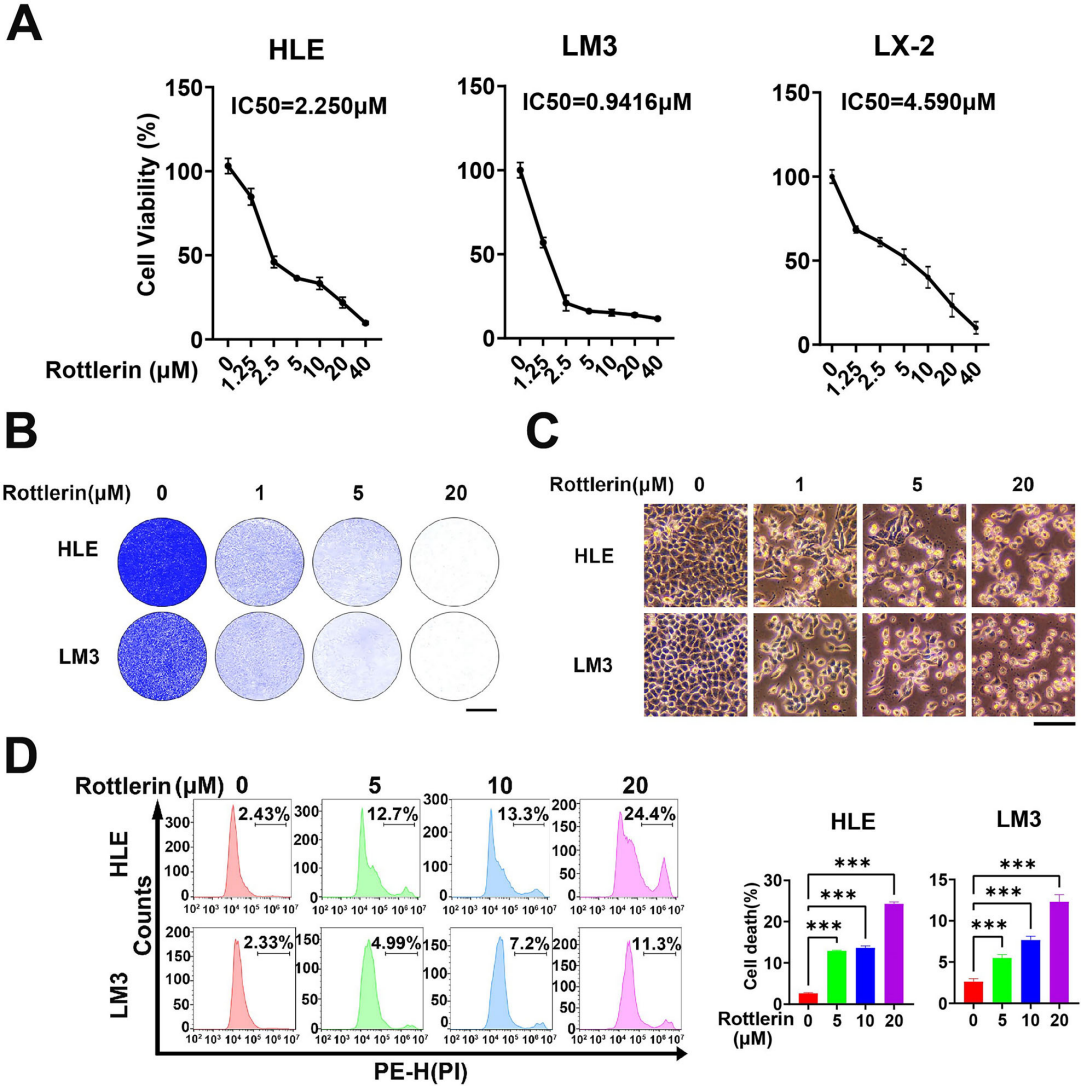
Rottlerin inhibits HCC cell growth. **A** HLE, LM3, and LX-2 cells were exposed to the indicated concentrations of rottlerin for 48 h. Cell viability was assessed using the CCK-8 assay, and the IC50 value was determined. Data were represented as mean ± SD (*n* = 4). **B** HLE and LM3 cells were treated with the specified concentrations of rottlerin for 52 h, then treated with crystal violet stain and photographed. Scale bar, 1 cm. **C** HLE and LM3 cells were treated with the specified concentrations of rottlerin for 72 h and then imaged under an inverted microscope. Scale bar, 100 μm. **D** HLE and LM3 cells were treated with the specified concentrations of rottlerin for 48 h, then stained with propidium iodide (PI) for flow cytometric analysis. The representative images (top) and cell death rates (bottom) were displayed. Data were represented as mean ± SD (*n* = 3) and analyzed using one-way ANOVA. ***P < 0.001.

To systematically evaluate the anti-proliferative effects of rottlerin in HCC, we treated HLE and LM3 cell lines with escalating concentrations of rottlerin (0–40 μM) and quantified viability using CCK-8 assays. Dose–response analyses demonstrated that rottlerin potently inhibited HCC cell proliferation, with half-maximal inhibitory concentrations (IC_50_) calculated as 2.250 μM for HLE cells and 0.9416 μM for LM3 cells (Fig. 1A). Importantly, the IC_50_ value of rottlerin in the normal hepatic stellate cells LX-2 (IC_50_ = 4.590 μM) was significantly higher than that in HLE and LM3 cells, highlighting the selective toxicity of rottlerin toward cancer cells (Fig. 1A). Consistent with these findings, crystal violet staining and bright-field microscopy revealed a progressive reduction in adherent cell density proportional to rottlerin concentration (0–20 μM), further confirming its dose-dependent growth-suppressive effects (Fig. 1B, C). To further validate rottlerin’s inhibitory effects on HCC cells, flow cytometry analyses were conducted on HLE and LM3 cell lines exposed to varying concentrations of rottlerin (0–20 μM) for 48 h. The results indicated that rottlerin-induced cell death was significantly increased in a dose-dependent manner (Fig. 1D). Notably, previous studies identified PKCδ as a putative rottlerin target with IC_50_ values ranging from 3 to 6 μM [22]. Given the sub-IC_50_ efficacy observed in our viability assays (LM3 IC_50_: 0.94 μM; HLE IC_50_: 2.25 μM), we selected 5 μM rottlerin for subsequent experiments to minimize off-target PKCδ inhibition while retaining robust anti-tumor activity, and 1 μM or lower rottlerin was classified as a low concentration.

### Rottlerin triggers ferroptotic cell death in HCC

**Fig. 2:**
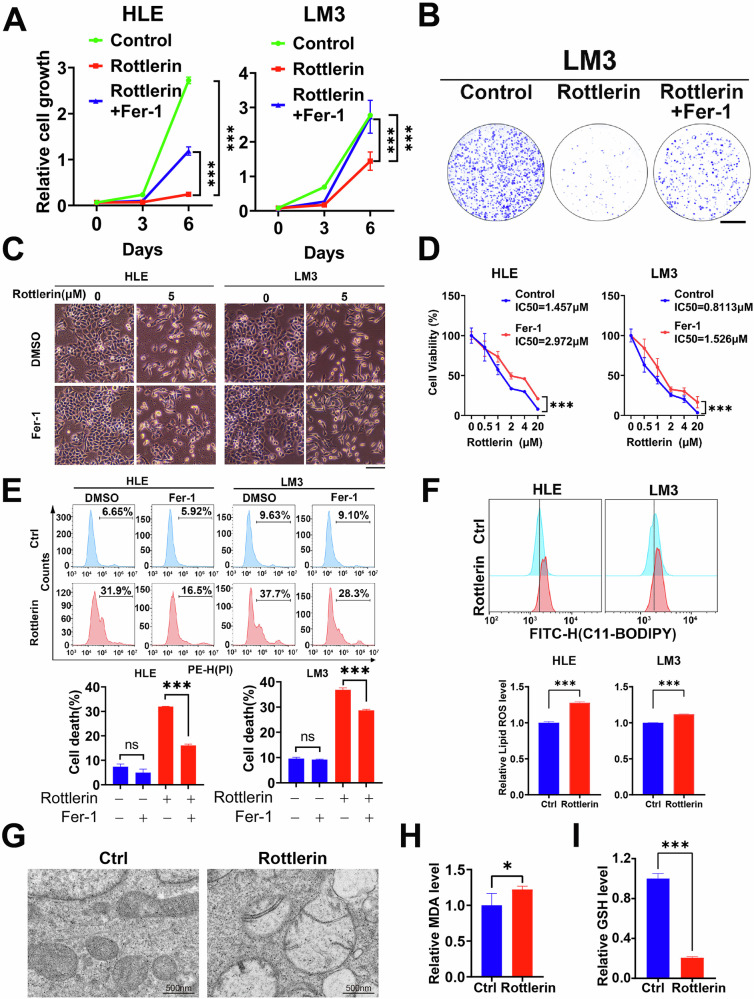
Rottlerin induces HCC cell ferroptosis. **A** HLE and LM3 cells were treated with 2 μM rottlerin or in combination with 2 μM Fer-1. The cells were stained with crystal violet and then dissolved with 10% glacial acetic acid. The absorbance at 595 nm was measured and calculated. Data were represented as mean ± SD (*n* = 3) and analyzed using Two-way ANOVA, ***P < 0.001. **B** LM3 cells were treated with 0.5 μM rottlerin with or without 2 μM Fer-1 for 14 days and then treated with crystal violet stain and photographed. Scale bar = 1 cm. **C** HLE and LM3 cells were treated with 5 μM rottlerin with or without 2 μM Fer-1 for 72 h and then imaged. Scale bar = 100 μm. **D** HLE and LM3 cells were exposed to the indicated doses of rottlerin with or without 2 μM Fer-1 for 48 h. Cell viability was assessed using the CCK-8 assay. Data were represented as mean ± SD (*n* = 3) and analyzed using two-way ANOVA, ***P < 0.001. **E** HLE and LM3 cells were treated with 5 μM rottlerin with or without 2 μM Fer-1 for 72 h, then stained with PI for flow cytometric analysis. The representative images (top) and cell death rates (bottom) were displayed. Data were represented as mean ± SD (*n* = 3) and analyzed using Student’s *t*-test; ns means not significant, ***P < 0.001. **F** HLE and LM3 cells were treated with 5 μM rottlerin for 60 h, then stained with the lipid ROS probe BODIPY 581/591 C11 for flow cytometric analysis. The representative images (top) and relative lipid ROS level (bottom) were displayed. Data were represented as mean ± SD (*n* = 3) and analyzed using Student’s *t*-test, ***P < 0.001. **G** The morphological changes of mitochondria were detected by transmission electron microscopy (TEM) in HLE cells incubated with or without 5 μM rottlerin for 48 h. Scale bar, 500 nm. **H** Measurement of malondialdehyde (MDA) levels in HLE cells treated with 5 μM rottlerin for 48 h. Data were represented as mean ± SD (*n* = 4) and analyzed using Student’s *t*-test, *P < 0.05. **I** Measurement of GSH levels in HLE cells treated with 5 μM rottlerin for 48 h. Data were represented as mean ± SD (*n* = 3) and analyzed using Student’s *t*-test, ***P < 0.001.

Ferroptosis is a newly identified programmed cell death characterized by excessive lipid peroxidation. Given that rottlerin can induce HCC cell death, we next investigated whether the rottlerin*-*mediated cell death of HCC cells was due to ferroptosis. HCC cells were treated with rottlerin in the absence or presence of ferroptosis inhibitor Ferrostatin-1 (Fer-1) [6]. We found that rottlerin significantly suppressed the growth of HLE and LM3 cells, and this suppression was rescued by Fer-1, suggesting ferroptosis as a primary contributor to rottlerin’s anti-tumor activity (Fig. 2A–C). Subsequently, cell viability assays demonstrated that Fer-1 could rescue cell death caused by rottlerin, and the IC_50_ of rottlerin significantly increased in the presence of Fer-1 (Fig. 2D). To mechanistically confirm this observation, flow cytometry analysis of propidium iodide (PI)-stained cells demonstrated that Fer-1 pretreatment reduced rottlerin-induced cell death in HLE and LM3 cells (Fig. 2E). Given that rottlerin can inhibit the growth of HCC cells and induce ferroptosis, we sought to determine whether rottlerin-induced ferroptosis directly contributes to this growth inhibition. To directly evaluate lipid peroxidation, a hallmark of ferroptosis, we quantified lipid ROS accumulation in HCC cells using the fluorescent lipid peroxidation sensor BODIPY 581/591 C11. Our results demonstrated that rottlerin significantly elevated lipid ROS levels in both HLE and LM3 cells, suggesting that rottlerin induces ferroptosis in HCC cells (Fig. 2F). Meanwhile, transmission electron microscopy was used to observe the structure of HLE cells under treatment with rottlerin. After rottlerin treatment, dramatic morphological changes of mitochondria were observed, including vacuolization, cristae enlargement, and fragmentation (Fig. 2G). Furthermore, we measured the levels of lipid peroxidation-derived malondialdehyde (MDA, a lipid peroxidation marker), glutathione (GSH), Fe^2+^, and PUFAs in HLE cells after rottlerin treatment. Compared to the control, the levels of MDA were elevated, and GSH levels were decreased in rottlerin-treated HLE cells (Fig. 2H and I). In contrast, there was no significant difference in the Fe^2+^ and PUFAs levels between rottlerin-treated HCC cells and the control group (Supplementary Fig. 1). These collective findings establish ferroptosis as the predominant cell death mechanism underlying rottlerin’s anti-proliferative effects in HCC.

### Low-dose rottlerin sensitizes HCC cells to ferroptosis

**Fig. 3:**
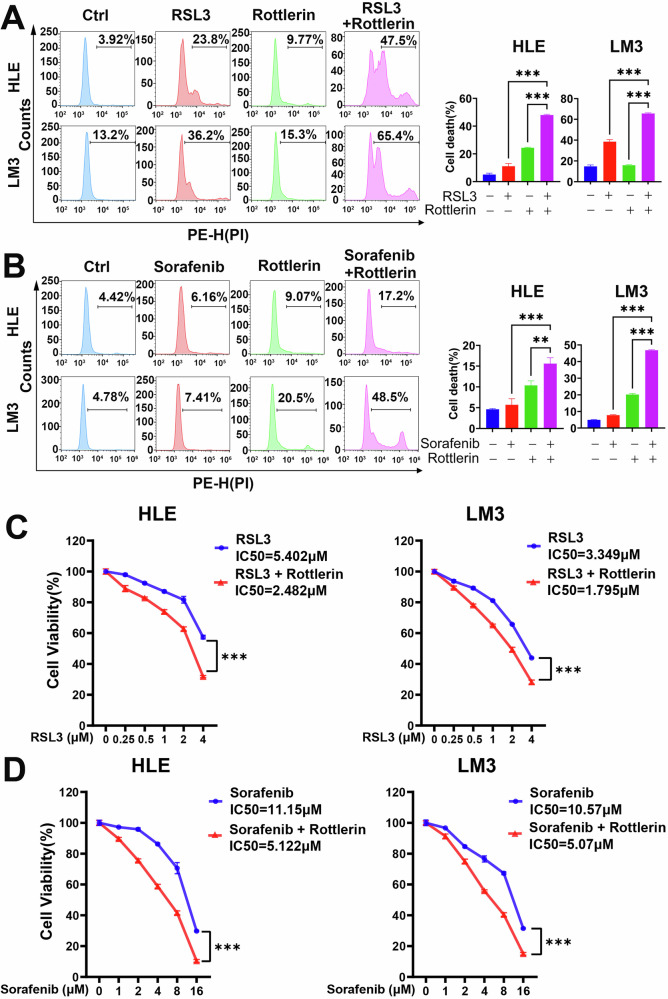
Low-dose rottlerin sensitizes HCC cells to ferroptosis. **A** 1 μM rottlerin and 5 μM RSL3 were added together in HLE and LM3 cells for 24 h, then stained with propidium iodide (PI) for flow cytometric analysis. The representative images (left) and cell death rates (right) were displayed. Data were represented as mean ± SD (*n* = 3) and analyzed using one-way ANOVA, ***P < 0.001. **B** 1 μM rottlerin and 10 μM sorafenib were added together in HLE and LM3 cells for 36 h, then stained with propidium iodide (PI) for flow cytometric analysis. The representative images (left) and cell death rates (right) were displayed. Data were represented as mean ± SD (*n* = 3) and analyzed using one-way ANOVA, **P < 0.01, ***P < 0.001. **C** HLE and LM3 cells were exposed to the indicated doses of RSL3 with or without 1 μM rottlerin for 48 h. Cell viability was assessed using the CCK-8 assay. Data were represented as mean ± SD (*n* = 3) and analyzed using Two-way ANOVA, ***P < 0.001. **D** HLE and LM3 cells were exposed to the indicated doses of sorafenib with or without 1 μM rottlerin for 48 h. Cell viability was assessed using the CCK-8 assay. Data were represented as mean ± SD (*n* = 3) and analyzed using Two-way ANOVA, ***P < 0.001.

RSL3 and sorafenib are effective ferroptosis inducers [35, 36]. Sorafenib is the drug approved for first-line systemic treatment of advanced HCC [37, 38]. However, increasing drug resistance has led to limited clinical responses and reduced therapeutic efficacy of sorafenib [39]. Combination therapy may be one of the effective strategies to overcome sorafenib resistance. We next investigated whether low concentrations of rottlerin (1 μM or lower rottlerin) can sensitize HCC cells to ferroptosis. Flow cytometry and cell viability assays were used to evaluate the effects of combining low concentrations of rottlerin with either RSL3 or sorafenib. Flow cytometry analysis showed that the cell death rate was significantly increased in the group treated with a low dose of rottlerin plus RSL3 or sorafenib compared to the groups treated with the same low concentration of rottlerin, RSL3, or sorafenib alone (Fig. 3A and B). Consistently, dose–response analyses demonstrated that the IC_50_ of RSL3 or sorafenib in HCC cells significantly decreased in the presence of the low-dose rottlerin (Fig. 3C and D). Altogether, these results indicated that even at low doses, rottlerin can sensitize HCC cells to ferroptosis, highlighting the potential of HCC treatment using the combination of rottlerin and canonical ferroptosis inducers.

### Rottlerin induces HCC cell ferroptosis not primarily dependent on PKCδ

**Fig. 4:**
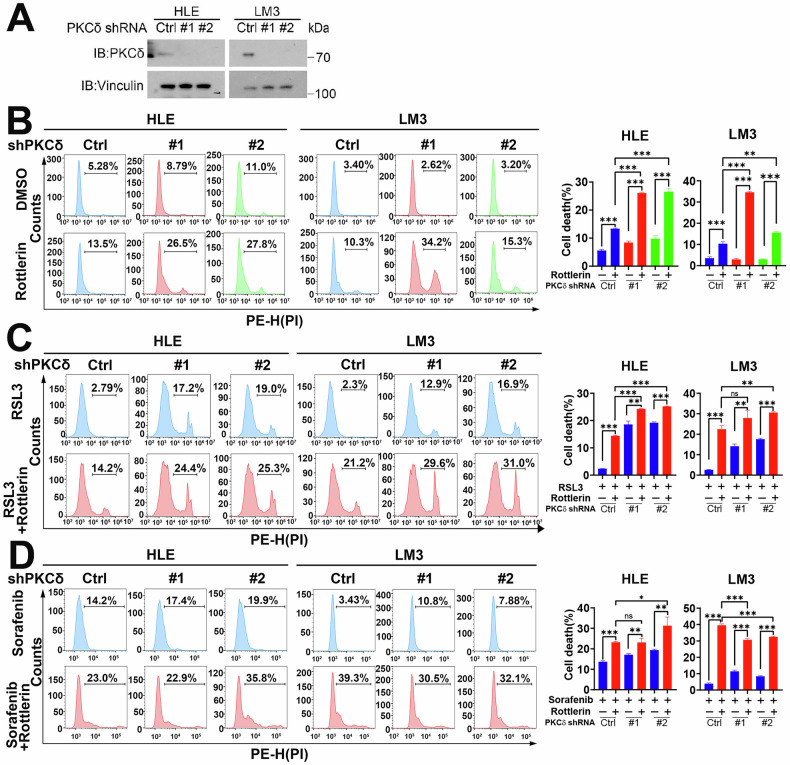
Rottlerin induces HCC cell ferroptosis not primarily dependent on PKCδ. **A** PKCδ protein levels were detected by Western blot. **B** 5 μM rottlerin was added in Control (shCtrl) and PKCδ-knockdown (sh#1, sh#2) HCC cells for 48 h, then cells were collected for PI staining and analyzed by flow cytometry. The representative images (left) and cell death rates (right) were displayed. Data were represented as mean ± SD (*n* = 3) and analyzed using one-way ANOVA, **P < 0.01, ***P < 0.001. **C** 2.5 μM RSL3 was added in Control (shCtrl) and PKCδ-knockdown (sh#1, sh#2) HCC cells with or without 1 μM rottlerin for 24 h, then cells were collected for PI staining and analyzed by flow cytometry. The representative images (left) and cell death rates (right) were displayed. Data were represented as mean ± SD (*n* = 3) and analyzed using one-way ANOVA, ns means not significant, **P < 0.01, ***P < 0.001. **D** 10 μM sorafenib was added in Control (shCtrl) and PKCδ-knockdown (sh#1, sh#2) HCC cells with or without 1 μM rottlerin for 36 h, then cells were collected for PI staining and analyzed by flow cytometry. The representative images (left) and cell death rates (right) were displayed. Data were represented as mean ± SD (*n* = 3) and analyzed using one-way ANOVA, ns means not significant, *P < 0.05, **P < 0.01, ***P < 0.001.

Research has indicated that rottlerin-induced autophagy and apoptosis occur independently of its target PKCδ [32]. To investigate whether rottlerin regulates HCC cell ferroptosis dependent on its canonical target PKCδ, the stable cell lines with PKCδ knockdown were constructed in HLE and LM3 cells. The efficiency of the knockdown was assessed using Western blot (Fig. 4A). Depletion of PKCδ (via knockdown or knockout) in HLE and HCCLM3 cells demonstrated that rottlerin treatment could still trigger hepatocellular carcinoma cell death, not primarily dependent on PKCδ (Fig. 4B and Supplementary Fig. 2). To assess whether the ferroptosis-enhancing effect of rottlerin depends on PKCδ, PKCδ-knockdown HLE and LM3 cells were treated with rottlerin in combination with the ferroptosis inducers RSL3 or sorafenib. Flow cytometry analysis revealed that the cell death rate in the PKCδ knockdown group treated with rottlerin and either RSL3 or sorafenib was significantly higher compared to the groups treated with RSL3 or sorafenib alone (Fig. 4C and D). These findings suggest that rottlerin’s ability to sensitize cells to ferroptosis is not primarily dependent on PKCδ. Even in the absence of PKCδ, low concentrations of rottlerin effectively enhanced ferroptosis induced by RSL3 or sorafenib in HCC cells.

### Rottlerin sensitizes HCC cells to ferroptosis via degrading SLC7A11 and GPX4

**Fig. 5:**
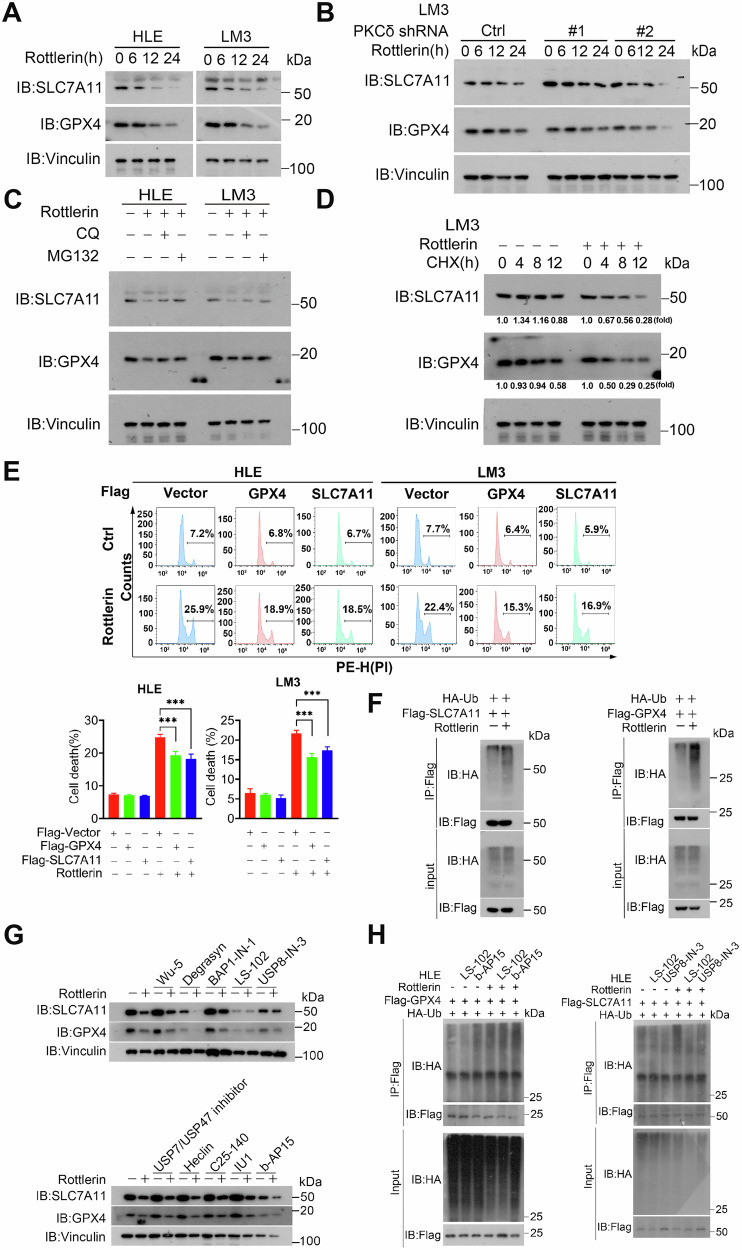
Rottlerin sensitizes HCC cells to ferroptosis by degrading SLC7A11 and GPX4. **A** HLE and LM3 cells were treated with 5 μM rottlerin for 0, 6, 12, and 24 h. The protein levels of GPX4 and SLC7A11 were detected by Western blot, with Vinculin as a loading control. **B** HLE and LM3 cells were pretreated with 5 μM rottlerin for 4 h, followed by the addition of 20 μM CQ or 10 μM MG132 for 6 h. GPX4 and SLC7A11 protein levels were detected by Western blot. **C** LM3 cells were pretreated with 5 μM rottlerin for 3 h, then treated with 100 μg/mL CHX for the indicated times. Cells were harvested and analyzed by Western blot. **D** Control (shCtrl) and PKCδ-knockdown (sh#1, sh#2) LM3 cells were treated with 5 μM rottlerin for 0, 6, 12, and 24 h. The protein levels of GPX4 and SLC7A11 were detected by Western blot. **E** HCC cells transfected with Flag-GPX4 or Flag-SLC7A11 were treated with or without 5 μM rottlerin for 48 h, then the cells were collected for PI staining and analyzed by flow cytometry. The representative images (Top) and cell death rates (Bottom) were displayed. Data were represented as mean ± SD (*n* = 3) and analyzed using one-way ANOVA, ***P < 0.001. **F** HLE cells were transfected with the indicated plasmids for 24 h and then treated with or without 5 μM rottlerin for 24 h. Cells were lysed for immunoprecipitation with anti-Flag antibodies, followed by Western blot with the specified antibodies. **G** HLE cells were pretreated with or without 10 μM Wu-5 (USP10 inhibitor), 10 μM Degrasyn (USP9x, USP5, USP14 and UCH37 inhibitor), 10 μM BAP1-IN-1 (BAP1 inhibitor), 10 μM LS-102 (SYVN1 inhibitor), 5 μM USP8-IN-3 (USP7/USP8 inhibitor), 5 μM USP7/USP47 inhibitor (USP7/USP47 inhibitor), 25 μM Heclin (HECT inhibitor), 20 μM C25-140 (TRAF6 inhibitor), 20 μM IU1 (USP14 inhibitor), and 5 μM b-AP15 (UCHL5/USP14 inhibitor) for 1 h, before being treated with or without 5 μM rottlerin for 24 h. Western blots were performed with the indicated antibodies. **H** HLE cells were transfected with the indicated plasmids for 24 h and then pretreated with or without 10 μM LS-102 (SYVN1 inhibitor), 5 μM b-AP15 (UCHL5 /USP14 inhibitor), and 5 μM USP8-IN-3 (USP7/USP8 inhibitor) for 1 h, before being treated with or without 5 μM rottlerin for 24 h. Cells were lysed for immunoprecipitation with anti-Flag antibodies, followed by Western blot with the specified antibodies.

Ferroptosis is a form of regulated cell death driven by iron-dependent phospholipid peroxidation. GPX4 (glutathione peroxidase 4) and SLC7A11 (a component of the system Xc-cystine/glutamate antiporter) play important roles in ferroptosis [40]. To understand the molecular mechanism of rottlerin*-*induced ferroptosis in HCC cells, HLE and LM3 cells were treated with 5 μM rottlerin for 6−24 h and analyzed using a Western blot experiment. The results showed that rottlerin significantly reduced the expression of SLC7A11 and GPX4 proteins over time (Fig. 5A). Furthermore, even after knocking down PKCδ, the GPX4 and SLC7A11 protein levels continued to decrease over time (Fig. 5B), implying that rottlerin regulates GPX4 expression not primarily dependent on its target PKCδ. To investigate the degradation pathways of SLC7A11 and GPX4 proteins, autophagy−lysosome inhibitor chloroquine (CQ) and ubiquitin−proteasome inhibitor MG132 were used. The results showed that in HLE and LM3 cells, MG132 was able to reverse the reduction in SLC7A11 and GPX4 protein levels caused by rottlerin, while CQ has no or minimal effect to rescue SLC7A11 or GPX4 protein reduction induced by rottlerin (Fig. 5C). To further explore the impact of rottlerin on protein stability of SLC7A11 and GPX4, LM3 cells were pretreated with rottlerin and then exposed to the protein synthesis inhibitor cycloheximide (CHX). Western blot analysis revealed that rottlerin significantly reduced the stability of both SLC7A11 and GPX4 proteins (Fig. 5D). These findings suggest that rottlerin mainly targets SLC7A11 and GPX4 for degradation through the ubiquitin−proteasome pathway. We further overexpressed SLC7A11/GPX4 in HCC cells to study the effect of rottlerin on ferroptosis. Flow cytometry analysis revealed that the cell death rate in the Flag-GPX4 or Flag-SLC7A11 group treated with rottlerin was significantly lower compared to the Flag-Vector group treated with rottlerin, suggesting rottlerin induced HCC cell death could be complemented by overexpressed SLC7A11/GPX4 (Fig. 5E). To further validate that rottlerin induces ferroptosis in HCC cells via degrading SLC7A11 and GPX4, we performed the immunoprecipitation assay. The results showed that rottlerin treatment did enhance the ubiquitination modification of SLC7A11 and GPX4 (Fig. 5F). To identify the specific components of the ubiquitin–proteasome system involved in rottlerin-induced degradation, we screened a panel of inhibitors targeting various E3 ubiquitin ligases and deubiquitinating enzymes previously implicated in the regulation of GPX4 and SLC7A11 [41, 42]. Treatment of HLE cells with these inhibitors revealed that LS-102, b-AP15, and USP8-IN-3—which inhibit SYVN1, the UCHL5/USP14 complex, and USP7/USP8, respectively—effectively prevented the rottlerin-induced reduction of both GPX4 and SLC7A11 protein levels (Fig. 5G). We next performed the immunoprecipitation assay to further investigate whether SYVN1, UCHL5/USP14, or USP7/USP8 directly mediates the ubiquitination of SLC7A11 and GPX4 in response to rottlerin. Notably, inhibition of the E3 ligase SYVN1 by LS-102 substantially suppressed rottlerin-mediated SLC7A11 ubiquitination (Fig. 5H), indicating that the E3 ubiquitin ligase SYVN1 may be the key ubiquitin-related factor involved in the ubiquitination and degradation of SLC7A11 induced by rottlerin. However, the key ubiquitin-related factor involved in the ubiquitination and degradation of GPX4 induced by rottlerin needs further investigation. These collective data indicate that rottlerin promotes the degradation of SLC7A11 and GPX4, contributing to ferroptosis induction in HCC cells.

### Combined treatment of rottlerin and sorafenib significantly inhibited HCC tumor progression

**Fig. 6:**
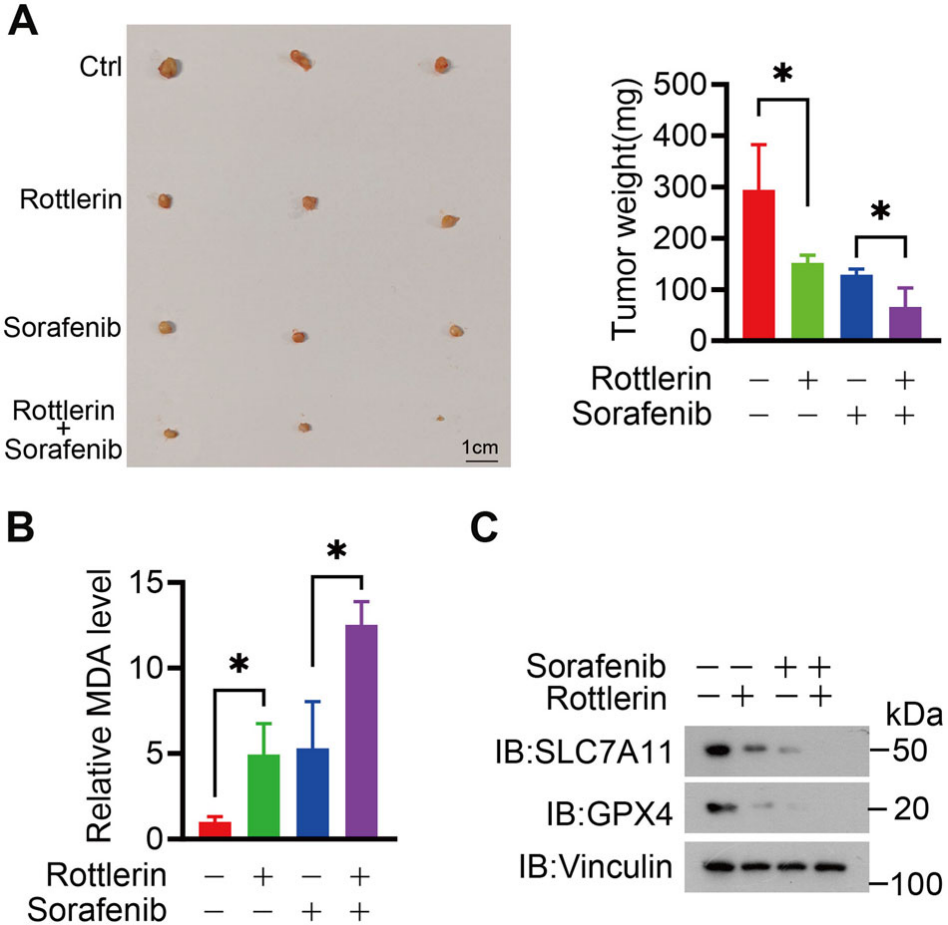
Treatment with rottlerin and sorafenib enhanced the inhibition of HCC tumor progression. **A** HLE cells line was subjected to mouse xenograft assays. After 10 days, the mice were assigned randomly into different treatment groups: treatment with rottlerin, with or without sorafenib. Tumor sizes were monitored and dissected tumors were weighed. Data were represented as mean ± SD (*n* = 3) and analyzed using Student’s *t*-test, *P < 0.05. **B** Measurement of malondialdehyde (MDA) levels of xenograft tumors from (**A**). Data were represented as mean ± SD (*n* = 3) and analyzed using Student’s *t*-test, *P < 0.05. **C** GPX4 and SLC7A11 protein levels of xenograft tumors from **A** were detected by Western blot.

To validate the effect of rottlerin on HCC tumor growth, we intraperitoneally injected rottlerin, with or without sorafenib, into HLE tumor-bearing nude mice. Consistent with the data derived from the in vitro experiments, rottlerin with sorafenib combination treatment resulted in markedly inhibited tumor progression along with an elevation in the MDA level compared with sorafenib treatment alone in the xenograft mice model (Fig. 6A and B). In addition, western blot analysis showed that the expression of SLC7A11 and GPX4 was decreased in the rottlerin-treated xenografts, indicating that rottlerin could induce ferroptosis in vivo. Moreover, the expression of SLC7A11 and GPX4 was the lowest in the rottlerin and sorafenib combined-treated xenograft tumors (Fig. 6C). These data collectively suggest that combining rottlerin with sorafenib significantly enhances their sensitivity to ferroptosis, rottlerin treatment significantly inhibited tumor growth in vivo, and dramatically increased the anti-tumor effect of sorafenib.

## Discussion

Liver cancer is one of the most common malignant tumors in China, with HCC accounting for 75–85% of primary liver cancer cases [43]. Primary liver cancer in China ranked fourth among common malignant tumors, and second in cancer-related death [44]. The years of life with disability caused by liver cancer in China account for nearly half of the global liver cancer burden [45]. Effectively reducing the burden of liver cancer remains a major challenge. The treatment goal for HCC is to improve survival rates and quality of life, but the high heterogeneity of tumors, ease of recurrence, and close association with chronic liver disease complicate treatment [46]. HCC patients often have varying degrees of liver function damage, limiting the choice of treatment methods, especially for therapeutic drugs with potential liver toxicity [47]. Ferroptosis is an iron-dependent form of programmed cell death that involves the regulation of multiple metabolic pathways and antioxidant systems. The immune response induced by ferroptosis in specific environments can be leveraged to treat primary liver tumors and liver metastases [48]. This study demonstrates that the natural product rottlerin inhibits the proliferation of HCC cells by inducing ferroptosis, offering new potential therapeutic avenues for liver cancer treatment. Previous research has shown that transcription factors such as the tumor suppressor BRCA1-associated protein 1 (BAP1) and activating transcription factor 3 (ATF3) reduce cystine uptake and enhance cell sensitivity to ferroptosis by inhibiting SLC7A11 expression; Additionally, malate dehydrogenase 2 (MDH2) can protect HCC cells from RSL3-induced ferroptosis via stabilizing GPX4 [49–51]. Several natural products, including auraptene and iberverin, have been reported to induce ferroptosis in hepatocellular carcinoma cells [52, 53]. In line with these findings, our study reveals that rottlerin promotes the degradation of both GPX4 and SLC7A11, thereby increasing HCC cell sensitivity to ferroptosis.

GPX4 has three isoforms: cytoplasmic (cGPX4), mitochondrial (mGPX4), and nuclear (nGPX4). Among them, cGPX4 is widely present in the cytoplasm and reduces cell death by resisting lipid peroxidation [11]. Previously, it was believed that only cGPX4 could prevent ferroptosis [54–56]. However, recent studies have shown that both cytoplasmic and mitochondrial GPX4 are essential for preventing ferroptosis in different subcellular compartments. Dihydroorotate dehydrogenase (DHODH) and mitochondrial GPX4 constitute two other defense enzymes that can detoxify lipid peroxides in mitochondria [57]. The potential role of nuclear GPX4 in regulating ferroptosis remains to be studied. In our study, we found that rottlerin acts on the SLC7A11−GSH−GPX4 axis, degrading SLC7A11 and GPX4, thereby inducing plasma membrane lipid peroxidation and ferroptosis. However, the specific mechanism by which rottlerin causes GPX4 and SLC7A11 degradation needs further investigation.

Rottlerin is a natural polyphenolic compound extracted from *Mallotus philippinensis* and serves as a specific inhibitor of PKC, capable of distinguishing between PKC isozymes with an IC50 value of 3−6 μM for PKC delta [22]. Rottlerin exerts its tumor suppressor function by inhibiting EZH2 expression in prostate cancer cells, thereby suppressing cell growth, migration, invasion, and inducing apoptosis [31]. Additionally, rottlerin induces apoptosis in pancreatic cancer cells [58], and induces autophagy, leading to apoptosis in breast cancer stem cells [30]. However, the relationship between rottlerin and ferroptosis has not been previously reported. In this study, we explored the relationship between rottlerin and ferroptosis in HCC and investigated the underlying mechanism, which provides a new treatment strategy for HCC. We found that rottlerin can induce ferroptosis in HCC by degrading SLC7A11 and GPX4, thereby inhibiting tumor growth. Additionally, we investigated whether rottlerin can sensitize cells to ferroptosis induced by RSL3 and sorafenib. RSL3 directly inhibits the activity of GPX4 and induces ferroptosis in tumor cells [35]. It can degrade GPX4 not only through the autophagy−lysosome pathway but also through the ubiquitin−proteasome system (UPS) [59], which is consistent with the GPX4 degradation pathway caused by rottlerin in our study.

Sorafenib is a multikinase inhibitor that promotes cell apoptosis, slows down angiogenesis, and inhibits tumor cell proliferation. Sorafenib acts on HCC cells, destroying the morphology of their mitochondria, accompanied by reduced oxidative phosphorylation activity, collapse of mitochondrial membrane potential, and reduced ATP synthesis, ultimately leading to cell death due to ferroptosis [37]. In advanced HCC, sorafenib is currently an effective first-line treatment. Rottlerin combined with sorafenib can also prevent T98G malignant glioma cells from being in the G1 phase [60]. Sorafenib induces ferroptosis in HCC by targeting SLC7A11 [36, 61]. Our results show that combining rottlerin with RSL3 or sorafenib significantly enhances their sensitivity to ferroptosis. Studies have shown that rottlerin’s ability to induce cell autophagy and apoptosis is independent of PKCδ [32]. We tested the effect of PKCδ knockdown on rottlerin-induced ferroptosis in HLE and LM3 and found that rottlerin’s regulation of ferroptosis in HCC cells was not affected by PKCδ knockdown. Therefore, rottlerin inhibits HCC progression by mediating ferroptosis through a mechanism that is not primarily dependent on PKCδ. It enhances the sensitivity of HCC cells to sorafenib and RSL3, thereby promoting ferroptosis. This process is facilitated by the degradation of GPX4 and SLC7A11, leading to increased ferroptosis and subsequent inhibition of HCC progression. Our findings suggest that rottlerin enhances sorafenib-induced ferroptosis in HCC, leading to enhanced inhibition of HCC tumor progression both in vitro and in vivo. It has been reported that TRIM7, HECTD3, TRIM3, and SOCS1 were found to bind to SLC7A11 and catalyze its ubiquitinated degradation [62–65]. So far, a few ubiquitin E3 ligases targeting GPX4, including TRIM21, TRIM26, TRIM46, and MARCHF1, have been reported [66–69]. Although we found that rottlerin targets the SLC7A11/GPX4 axis for ubiquitin–proteasomal degradation, driving ferroptosis in HCC cells. However, the specific ubiquitin E3 ligase and ubiquitination sites of SLC7A11 and GPX4 remain to be explored in the future. Our research might provide new options for the treatment of HCC, which will help reduce the burden of HCC treatment and improve the quality of life for patients.

## Materials and methods

### Cell lines and cell culture

Human hepatocellular carcinoma cell lines HLE and HCCLM3 (LM3), normal hepatic stellate cells (LX-2), along with the human embryonic kidney cell line (HEK-293T), were obtained from the Cell Bank of the Chinese Academy of Sciences. The cells were cultured in high-glucose Dulbecco’s Modified Eagle Medium (DMEM, Solarbio, Cat# 11965, China) supplemented with 10% fetal bovine serum (FBS, Excell Bio, Cat# FSP500, China), 100 U/mL penicillin, and 100 μg/mL streptomycin. All cells were maintained in a humidified incubator at 37 °C with 5% CO₂.

### Regents and antibodies

The reagents used in this study are as follows: Rottlerin (TargetMol, Cat# T16791, USA); RSL3 (CSNpharm, Cat# CSN17581, USA); Sorafenib (Selleck, Cat# S7397, USA); Ferrostatin-1 (Fer-1, TargetMol, Cat# T6500, USA); C11−BODIPY 581/591 (MCE, Cat# HY-D1691, China); Propidium Iodide (PI, TargetMol, Cat# T2130, USA); 0.25% trypsin digestion solution (Servicebio, Cat# G4012, China); Dimethyl sulfoxide (DMSO, Solarbio, Cat# D8371, China); Cell lysis buffer (CST, Cat# 9803S, USA), PMSF (Wuhan Dingguo Biotechnology, Cat# 329-98-6, China); MG132 (Biovision, Cat# 1791-5, USA); Chloroquine (CQ, MCE, Cat# HY-17589A, China); Cycloheximide (CHX, MCE, Cat# HY-12320, China); Paraformaldehyde (Solarbio, Cat# P1110, China); Sodium dodecyl sulfate (SDS, Solarbio, Cat# S8010, China). GSH test kit (Servicebio, Cat# G4305-48T, China). Enzyme-linked immunosorbent assay (ELISA) kit (MEIMIAN, Cat# MM-92625102, China). USP7/USP47 inhibitor (KKL Med, Cat# 1247825-37-1, USA), Heclin (KKL Med, Cat# 890605-54-6, USA), C25-140 (KKL Med, Cat# 1358099-18-9, USA), IU1 (KKL Med, Cat# 314245-33-5, USA), b-AP15 (KKL Med, Cat# 1009817-63-3, USA), Wu-5 (TargetMol, Cat# 2630378-05-9, China), Degrasyn (TargetMol, Cat# 856243-80-6, China), BAP1-IN-1 (TargetMol, Cat# 353495-21-3, China), LS-102 (Ambeed, Cat# A1340566, China) and USP8-IN-3 (KKL Med, Cat# 2477651-10-6, USA).

Antibodies used in this study are as follows: Vinculin (Santa Cruz, Cat# sc-73614, USA, used at 1:20,000); SLC7A11 (Proteintech, Cat# 18790-1-AP, China, used at 1:3000); GPX4 (Proteintech, Cat# 67763-1-Ig, China, used at 1:2000); Peroxidase-AffiniPure goat anti-mouse IgG(H + L) (Jackson Immunoresearch, Cat# 115-035-003, USA, used at 1:10,000); Peroxidase IgG Fraction Monoclonal Mouse Anti-Rabbit IgG, light chain specific (Jackson Immunoresearch, USA, Cat# 211-032-171, used at 1:10,000).

### Cell death analysis

Cell death was assessed through propidium iodide (PI) staining. Cells were harvested via enzymatic digestion, washed with PBS three times, and stained with 10 μg/mL PI for at least 15 min at room temperature in the dark. Following staining, the cells were centrifuged at 2000 rpm for 3 min, washed with PBS, resuspended in 500 μL of PBS, and maintained on ice for flow cytometry analysis. The resulting data were processed using FlowJo software.

### Cell viability assay

Cell viability was evaluated using the Cell Counting Kit-8 (CCK-8, MeilunBio, Cat# MA0218, China). HLE and LM3 cells were digested, counted, and seeded at a density of 3000 cells per well in 200 μL of DMEM within 96−well plates. After cell adherence, rottlerin and Fer-1 were added as outlined in the experimental protocol. Following the treatment period, 20 μL of CCK-8 reagent was introduced to each well, and the plates were incubated at 37 °C for 1 h in the dark. The optical density (OD) at 450 nm was measured using a microplate reader to determine the cell survival rate.

### Cell proliferation assay

HLE and LM3 cells (3000 cells per well) were plated in 24-well plates. After cell adherence, the cells were treated with specified concentrations of rottlerin and Fer-1 for 3–6 days. Following treatment, the cells were washed with PBS, fixed with 4% paraformaldehyde (Solarbio, Cat# P1110, China) at room temperature for 30 min, and washed three more times with PBS. The cells were then stained with 500 μL of crystal violet for 5 min, and excess stain was removed by washing. To quantify staining, 200 μL of 10% glacial acetic acid was added to dissolve the crystal violet, and the solution was shaken and eluted for 1 h. The dissolved crystal violet solution was transferred to 96−well plates, and the optical density (OD) at 595 nm was measured using a microplate reader to generate the cell growth curve.

### Colony formation assay

HLE and LM3 cells were plated in six-well plates at a density of 2000 cells per well and cultured for 14 days. After cell adherence, specified concentrations of rottlerin and Fer-1 were added, and the medium was refreshed every 3 days. At the end of the culture period, the medium was removed, and the cells were washed three times with PBS. The cells were then fixed with 4% paraformaldehyde for 30 min at room temperature and stained with 0.1% crystal violet containing 20% methanol for 5 min. Afterward, the cells were washed with PBS and photographed.

### Western blot

HLE and LM3 cells were lysed at 4 °C for 30 min using a lysis buffer (CST, Cat# 9803S, USA) supplemented with 1% protease inhibitor PMSF (Wuhan Dingguo Biotechnology, Cat# 329-98-6, China). The lysates were centrifuged at 12,000 rpm for 15 min at 4 °C, and the supernatant was collected. An equal volume of 2× SDS loading buffer was added, and the mixture was boiled for 10 min. The samples were then separated by SDS−PAGE and transferred to PVDF membranes (Millipore, Cat# 03010040001, USA). The membranes were blocked with 5% skim milk powder (Solarbio, Cat# D8340, China) at room temperature for 1 h, and then incubated with the specified antibodies. After washing with TBST, protein bands were visualized using the ECL chemiluminescence kit (Meilunbio, Cat# MA0186, China).

### Lentiviral transfection

For stable knockdown of PKCδ, shPKCδ plasmids were constructed. The shRNA sequences targeting PKCδ were listed below: shPKCδ #1: CAACAGCCGGGACACTATATT; shPKCδ #2: TCAGAGCCTGTTGGGATATAT. Briefly, HEK-293T cells were grown to 80% confluency and then co-transfected with the target plasmid (plko.1 or shPKCδ plasmid) along with the lentiviral packaging plasmids PSPAX2 and PMD2.G. After 48 h, the supernatant was harvested and filtered through a 0.45 μm filter to remove cellular debris. The filtered supernatant, supplemented with 5 μg/mL polybrene (MCE, Cat# HY-112735, China), was used to infect HLE and LM3 cells. After 48 h of infection, 2 μg/mL puromycin (Solarbio, Cat# P8230, China) was added to the culture medium to eliminate uninfected cells.

### Detection of intracellular lipid ROS and Fe^2+^ levels

HLE and LM3 cells were plated in 12-well plates, and 5 μM rottlerin was added after cell adhesion. Following incubation in the dark for the specified duration, the medium was replaced with PBS containing the lipid ROS probe BODIPY 581/591 C11 (MCE, Cat# HY-D1691, China) or Fe^2+^ probe RhoNox-1 (TargetMol, Cat# T87330, China), and the cells were incubated at 37 °C for 30 min. The cells were then collected, washed, resuspended in 500 μL of PBS, and analyzed using flow cytometry. Data analysis was conducted using FlowJo software.

### Transmission electron microscope (TEM)

After indicated treatment, HLE cells were fixed with the 2.5% glutaraldehyde solution at 4 °C overnight. After fixation, the samples were dehydrated by a graded series of ethanol, then dehydrated by alcohol and eventually transferred to absolute acetone. Following infiltration with absolute acetone and the final Spurr resin mixture, the samples were embedded, ultrathin sectioned, and stained. Finally, the samples were observed in the Hitachi Model H-7650 transmission electron microscope.

### MDA assay

The levels of lipid peroxidation product MDA in cells or tumor tissues were determined by Micro MDA assay Kit (Nanjing Jiancheng Bioengineering Institute, China) according to the manufacturer’s instructions. The tumor tissues were ground, and the cells were lysed. Briefly, cell lysates were incubated with thiobarbituric acid (TBA) at 95 °C for 80 min. After cooling to room temperature, the absorbance at 532 nm was determined using a microplate reader.

### Intracellular glutathione (GSH) assay

Intracellular glutathione (GSH) assay was performed as described previously [52]. Briefly, HLE cells were treated with 5 μM rottlerin for 48 h. Then, the cells were collected and lysed. The supernatant with or without the rottlerin treatment was mixed with the detection probe working solution and incubated at room temperature for 5 min before measurement of the absorbance at 412 nm.

### Ubiquitination assay

Ubiquitination assay was performed following the procedure described previously [70]. Briefly, HLE cells were transiently transfected with plasmids (HA-Ub, Flag-SLC7A11, or Flag-GPX4). After 24 h of transfection, cells were treated with 5 μM rottlerin for 24 h, and then cells were harvested and lysed with cell lysis buffer (CST, Cat# 9803S, USA) supplemented with 1 mM PMSF and 20 mM N-ethylmaleimide (NEM). Then, immunoprecipitation was performed with anti-Flag antibodies. Immunoprecipitated proteins were analyzed by Western blot using the indicated antibodies.

### Xenograft mice model

All xenograft experiments were approved by the Animal Ethics Committee of Nanchang University. Briefly, male 5- to 6-week-old athymic BALB/c nude mice were purchased from GemPharmatech Co., Ltd (Nanjing, China). Mice were randomly assigned to experimental groups and 5 × 10^6^ HLE cells in PBS (100 μL) were injected subcutaneously into the nude mice. After 10 days, the mice were randomly assigned to four groups for the indicated treatments. Rottlerin was injected intraperitoneally into mice at a dose of 5 mg/kg every 2 days. Simultaneously, sorafenib was injected intraperitoneally at a dose of 20 mg/kg every 2 days. Three weeks after the injection of HLE cells, the mice were euthanized. Then, the tumor tissues were photographed and weighed. Formal synergy modeling was not performed in this study.

### Statistical analysis

Statistical analyses were performed using GraphPad Prism 9. Results are expressed as mean ± standard deviation (SD). Comparisons between two groups were analyzed using Student’s *t*-test, while comparisons among multiple groups were evaluated using ANOVA. *P*-value of <0.05 was considered statistically significant.

## Supplementary information

Supplementary figures and figure legends

uncropped original western blots

## Data Availability

All data and materials of this study are available from the corresponding author upon reasonable request.

## References

[1] Bian XL, Chen HZ, Yang PB, Li YP, Zhang FN, Zhang JY, et al. Nur77 suppresses hepatocellular carcinoma via switching glucose metabolism toward gluconeogenesis through attenuating phosphoenolpyruvate carboxykinase sumoylation. Nat Commun. 2017;8:14420. 28240261 10.1038/ncomms14420 PMC5333363

[2] Hou PP, Luo LJ, Chen HZ, Chen QT, Bian XL, Wu SF, et al. Ectosomal PKM2 promotes HCC by inducing macrophage differentiation and remodeling the tumor microenvironment. Mol Cell. 2020;78:1192–1206.e1110. 32470318 10.1016/j.molcel.2020.05.004

[3] Bray F, Laversanne M, Sung H, Ferlay J, Siegel RL, Soerjomataram I, et al. Global cancer statistics 2022: GLOBOCAN estimates of incidence and mortality worldwide for 36 cancers in 185 countries. CA: Cancer J Clin. 2024;74:229–63. 38572751 10.3322/caac.21834

[4] Han B, Zheng R, Zeng H, Wang S, Sun K, Chen R, et al. Cancer incidence and mortality in China, 2022. J Natl Cancer Center. 2024;4:47–53. 39036382 10.1016/j.jncc.2024.01.006 PMC11256708

[5] Li Q, Peng F, Yan X, Chen Y, Zhou J, Wu S, et al. Inhibition of SLC7A11-GPX4 signal pathway is involved in aconitine-induced ferroptosis in vivo and in vitro. J Ethnopharmacol. 2023;303:116029. 36503029 10.1016/j.jep.2022.116029

[6] Dixon SJ, Lemberg KM, Lamprecht MR, Skouta R, Zaitsev EM, Gleason CE, et al. Ferroptosis: an iron-dependent form of nonapoptotic cell death. CELL. 2012;149:1060–72. 22632970 10.1016/j.cell.2012.03.042 PMC3367386

[7] Harris IS, DeNicola GM. The complex interplay between antioxidants and ROS in cancer. Trends Cell Biol. 2020;30:440–51. 32303435 10.1016/j.tcb.2020.03.002

[8] Badgley MA, Kremer DM, Maurer HC, DelGiorno KE, Lee H-J, Purohit V, et al. Cysteine depletion induces pancreatic tumor ferroptosis in mice. Science. 2020;368:85–9. 32241947 10.1126/science.aaw9872 PMC7681911

[9] Koppula P, Zhang Y, Zhuang L, Gan B. Amino acid transporter SLC7A11/xCT at the crossroads of regulating redox homeostasis and nutrient dependency of cancer. Cancer Commun (Lond). 2018;38:12. 29764521 10.1186/s40880-018-0288-x PMC5993148

[10] Forman HJ, Zhang H, Rinna A. Glutathione: overview of its protective roles, measurement, and biosynthesis. Mol Aspects Med. 2009;30:1–12. 18796312 10.1016/j.mam.2008.08.006 PMC2696075

[11] Liu Y, Wan Y, Jiang Y, Zhang L, Cheng W. GPX4: the hub of lipid oxidation, ferroptosis, disease and treatment. Biochim Biophys Acta Rev Cancer. 2023;1878:188890. 37001616 10.1016/j.bbcan.2023.188890

[12] Huang B, Wang H, Liu S, Hao M, Luo D, Zhou Y, et al. Palmitoylation-dependent regulation of GPX4 suppresses ferroptosis. Nat Commun. 2025;16:867. 39833225 10.1038/s41467-025-56344-5 PMC11746948

[13] Xu T, Ding W, Ji X, Ao X, Liu Y, Yu W, et al. Molecular mechanisms of ferroptosis and its role in cancer therapy. J Cell Mol Med. 2019;23:4900–12. 31232522 10.1111/jcmm.14511 PMC6653007

[14] Chen X, Comish PB, Tang D, Kang R. Characteristics and biomarkers of ferroptosis. Front Cell Dev Biol. 2021;9:637162. 33553189 10.3389/fcell.2021.637162 PMC7859349

[15] Lei G, Zhuang L, Gan B. Targeting ferroptosis as a vulnerability in cancer. Nat Rev Cancer. 2022;22:381–96. 35338310 10.1038/s41568-022-00459-0 PMC10243716

[16] Mellor H, Parker PJ. The extended protein kinase C superfamily. Biochem J. 1998;332:281–92. 9601053 10.1042/bj3320281 PMC1219479

[17] Steinberg SF. Distinctive activation mechanisms and functions for protein kinase Cdelta. Biochem J. 2004;384:449–59. 15491280 10.1042/BJ20040704 PMC1134130

[18] Shin EJ, Hwang YG, Sharma N, Tran HQ, Dang DK, Jang CG, et al. Role of protein kinase Cdelta in dopaminergic neurotoxic events. Food Chem Toxicol. 2018;121:254–61. 30195712 10.1016/j.fct.2018.09.005

[19] Lonne GK, Masoumi KC, Lennartsson J, Larsson C. Protein kinase Cdelta supports survival of MDA-MB-231 breast cancer cells by suppressing the ERK1/2 pathway. J Biol Chem. 2009;284:33456–65. 19833733 10.1074/jbc.M109.036186 PMC2785190

[20] Yamada K, Oikawa T, Kizawa R, Motohashi S, Yoshida S, Kumamoto T, et al. Unconventional secretion of PKCdelta exerts tumorigenic function via stimulation of ERK1/2 signaling in liver cancer. Cancer Res. 2021;81:414–25. 33318039 10.1158/0008-5472.CAN-20-2009

[21] Mandal JP, Shiue CN, Chen YC, Lee MC, Yang HH, Chang HH, et al. PKCdelta mediates mitochondrial ROS generation and oxidation of HSP60 to relieve RKIP inhibition on MAPK pathway for HCC progression. Free Radic Biol Med. 2021;163:69–87. 33307168 10.1016/j.freeradbiomed.2020.12.003

[22] Gschwendt M, Muller HJ, Kielbassa K, Zang R, Kittstein W, Rincke G, et al. Rottlerin, a novel protein kinase inhibitor. Biochem Biophys Res Commun. 1994;199:93–8. 8123051 10.1006/bbrc.1994.1199

[23] Ma J, Hou Y, Xia J, Zhu X, Wang ZP. Tumor suppressive role of rottlerin in cancer therapy. Am J Transl Res. 2018;10:3345–56. 30662591 PMC6291697

[24] Yang MH, Kim J, Khan IA, Walker LA, Khan SI. Nonsteroidal anti-inflammatory drug activated gene-1 (NAG-1) modulators from natural products as anti-cancer agents. Life Sci. 2014;100:75–84. 24530873 10.1016/j.lfs.2014.01.075

[25] Tripathi N, Mandrah K, Goel B, Bhardwaj N, Paswan VK, Ravikanth G, et al. In-vitro anti-inflammatory potential of standardized rottlerin enriched fraction of *Mallotus philippensi*s Muell. Arg anti-inflammatory potential of rottlerin enriched fraction of *Mallotus philippensis*. Proc Natl Acad Sci India Sect B: Biol Sci. 2024;94:389–95.

[26] Ojha D, Winkler CW, Leung JM, Woods TA, Chen CZ, Nair V, et al. Rottlerin inhibits La Crosse virus-induced encephalitis in mice and blocks release of replicating virus from the Golgi body in neurons. Nat Microbiol. 2021;6:1398–409. 34675384 10.1038/s41564-021-00968-y

[27] Zhou S, Lin Q, Huang C, Luo X, Tian X, Liu C, et al. Rottlerin plays an antiviral role at early and late steps of Zika virus infection. Virol Sin. 2022;37:685–94. 35934227 10.1016/j.virs.2022.07.012 PMC9583117

[28] Hwang Y, Kim HC, Shin EJ. Enhanced neurogenesis is involved in neuroprotection provided by rottlerin against trimethyltin-induced delayed apoptotic neuronal damage. Life Sci. 2020;262:118494. 32991881 10.1016/j.lfs.2020.118494

[29] Kim YJ, Go G-W. Rottlerin suppresses fat accumulation by inhibiting adipogenesis and de novo lipogenesis in 3T3-L1 adipocytes. Current Dev Nutr. 2021;5:1224. 10.1007/s10068-023-01339-5 PMC10349001 37457404

[30] Kumar D, Shankar S, Srivastava RK. Rottlerin-induced autophagy leads to the apoptosis in breast cancer stem cells: molecular mechanisms. Mol Cancer. 2013;12:171. 24359639 10.1186/1476-4598-12-171 PMC3914415

[31] Zheng N, Wang L, Hou Y, Zhou X, He Y, Wang Z. Rottlerin inhibits cell growth and invasion via down-regulation of EZH2 in prostate cancer. Cell Cycle. 2018;17:2460–73. 30394832 10.1080/15384101.2018.1542897 PMC6342076

[32] Song KS, Kim JS, Yun EJ, Kim YR, Seo KS, Park JH, et al. Rottlerin induces autophagy and apoptotic cell death through a PKC-delta-independent pathway in HT1080 human fibrosarcoma cells: the protective role of autophagy in apoptosis. Autophagy. 2008;4:650–8. 18424913 10.4161/auto.6057

[33] Erdogan MA, Yilmaz OA. Rottlerin and genistein inhibit neuroblastoma cell proliferation and invasion through EF2K suppression and related protein pathways. Naunyn Schmiedebergs Arch Pharmacol. 2023;396:2481–2500. 37083712 10.1007/s00210-023-02473-x

[34] Shi J, Ning H, He G, Huang Y, Wu Z, Jin L, et al. Rottlerin inhibits cell growth, induces apoptosis and cell cycle arrest, and inhibits cell invasion in human hepatocellular carcinoma. Mol Med Rep. 2018;17:459–64. 29115596 10.3892/mmr.2017.7924

[35] Sui X, Zhang R, Liu S, Duan T, Zhai L, Zhang M, et al. RSL3 drives ferroptosis through GPX4 inactivation and ROS production in colorectal cancer. Front Pharmacol. 2018;9:1371. 30524291 10.3389/fphar.2018.01371 PMC6262051

[36] Shi Z, Li Z, Jin B, Ye W, Wang L, Zhang S, et al. Loss of LncRNA DUXAP8 synergistically enhanced sorafenib induced ferroptosis in hepatocellular carcinoma via SLC7A11 de-palmitoylation. Clin Transl Med. 2023;13:e1300. 37337470 10.1002/ctm2.1300 PMC10280000

[37] Li Y, Xia J, Shao F, Zhou Y, Yu J, Wu H, et al. Sorafenib induces mitochondrial dysfunction and exhibits synergistic effect with cysteine depletion by promoting HCC cells ferroptosis. Biochem Biophys Res Commun. 2021;534:877–84. 33162029 10.1016/j.bbrc.2020.10.083

[38] Llovet JM, Ricci S, Mazzaferro V, Hilgard P, Gane E, Blanc JF, et al. Sorafenib in advanced hepatocellular carcinoma. N Engl J Med. 2008;359:378–90. 18650514 10.1056/NEJMoa0708857

[39] Shi Y, Yang X, Xue X, Sun D, Cai P, Song Q, et al. HANR enhances autophagy-associated sorafenib resistance through miR-29b/ATG9A axis in hepatocellular carcinoma. OncoTargets Ther. 2020;13:2127–37. 10.2147/OTT.S229913 PMC7069583 32210579

[40] Jiang X, Stockwell BR, Conrad M. Ferroptosis: mechanisms, biology and role in disease. Nat Rev Mol Cell Biol. 2021;22:266–82. 33495651 10.1038/s41580-020-00324-8 PMC8142022

[41] Meng Y, Sun H, Li Y, Zhao S, Su J, Zeng F, et al. Targeting ferroptosis by ubiquitin system enzymes: a potential therapeutic strategy in cancer. Int J Biol Sci. 2022;18:5475–88. 36147464 10.7150/ijbs.73790 PMC9461661

[42] Zhu L, Liu YP, Yuan W, Sun BX, Huang YT, Zhao JK, et al. E3 ubiquitin ligase SYVN1 as a promising therapeutic target for diverse human diseases. Pharmacol Res. 2025;212:107603. 39818260 10.1016/j.phrs.2025.107603

[43] Zhou M, Wang H, Zeng X, Yin P, Zhu J, Chen W, et al. Mortality, morbidity, and risk factors in China and its provinces, 1990–2017: a systematic analysis for the Global Burden of Disease Study 2017. Lancet. 2019;394:1145–58. 31248666 10.1016/S0140-6736(19)30427-1 PMC6891889

[44] Zhou J, Sun H, Wang Z, Cong W, Zeng M, Zhou W, et al. Guidelines for the diagnosis and treatment of primary liver cancer (2022 Edition). Liver Cancer. 2023;12:405–44. 37901768 10.1159/000530495 PMC10601883

[45] Shi JF, Cao M, Wang Y, Bai FZ, Lei L, Peng J, et al. Is it possible to halve the incidence of liver cancer in China by 2050?. Int J Cancer. 2021;148:1051–65. 32997794 10.1002/ijc.33313

[46] Llovet JM, Kelley RK, Villanueva A, Singal AG, Pikarsky E, Roayaie S, et al. Hepatocellular carcinoma. Nat Rev Disease Primers. 2021;7:6. 33479224 10.1038/s41572-020-00240-3 PMC13537433

[47] Llovet JM, Pinyol R, Kelley RK, El-Khoueiry A, Reeves HL, Wang XW, et al. Molecular pathogenesis and systemic therapies for hepatocellular carcinoma. Nat Cancer. 2022;3:386–401. 35484418 10.1038/s43018-022-00357-2 PMC9060366

[48] Conche C, Finkelmeier F, Pesic M, Nicolas AM, Boettger TW, Kennel KB, et al. Combining ferroptosis induction with MDSC blockade renders primary tumours and metastases in liver sensitive to immune checkpoint blockade. GUT. 2023;72:1774–82. 36707233 10.1136/gutjnl-2022-327909 PMC10423492

[49] Zhang Y, Shi J, Liu X, Feng L, Gong Z, Koppula P, et al. BAP1 links metabolic regulation of ferroptosis to tumour suppression. Nat Cell Biol. 2018;20:1181–92. 30202049 10.1038/s41556-018-0178-0 PMC6170713

[50] Dai E, Meng L, Kang R, Wang X, Tang D. ESCRT-III-dependent membrane repair blocks ferroptosis. Biochem Biophys Res Commun. 2020;522:415–21. 31761326 10.1016/j.bbrc.2019.11.110 PMC6957708

[51] Yu W, Li Y, Gao C, Li D, Chen L, Dai B, et al. MDH2 promotes hepatocellular carcinoma growth through ferroptosis evasion via stabilizing GPX4. Int J Mol Sci. 2024;25:11604. 39519171 10.3390/ijms252111604 PMC11546247

[52] Li D, Li Y, Chen L, Gao C, Dai B, Yu W, et al. Natural product auraptene targets SLC7A11 for degradation and induces hepatocellular carcinoma ferroptosis. Antioxidants (Basel). 2024;13:1015. 39199259 10.3390/antiox13081015 PMC11351406

[53] Yang H, Dai B, Chen L, Li Y, Jin X, Gao C, et al. Iberverin downregulates GPX4 and SLC7A11 to induce ferroptotic cell death in hepatocellular carcinoma cells. Biomolecules. 2024;14:1407. 39595583 10.3390/biom14111407 PMC11592392

[54] Yant LJ, Ran Q, Rao L, Van Remmen H, Shibatani T, Belter JG, et al. The selenoprotein GPX4 is essential for mouse development and protects from radiation and oxidative damage insults. Free Radic Biol Med. 2003;34:496–502. 12566075 10.1016/s0891-5849(02)01360-6

[55] Conrad M, Moreno SG, Sinowatz F, Ursini F, Kölle S, Roveri A, et al. The nuclear form of phospholipid hydroperoxide glutathione peroxidase is a protein thiol peroxidase contributing to sperm chromatin stability. Mol Cell Biol. 2005;25:7637–44. 16107710 10.1128/MCB.25.17.7637-7644.2005 PMC1190272

[56] Liang H, Yoo S-E, Na R, Walter C, Richardson A, Ran Q. Short form glutathione peroxidase 4 is the essential isoform required for survival and somatic mitochondrial functions. J Biol Chem. 2009;284:30836–44. 19744930 10.1074/jbc.M109.032839 PMC2781482

[57] Mao C, Liu X, Zhang Y, Lei G, Yan Y, Lee H, et al. DHODH-mediated ferroptosis defence is a targetable vulnerability in cancer. Nature. 2021;593:586–90. 33981038 10.1038/s41586-021-03539-7 PMC8895686

[58] Su JN, Wang LX, Yin XY, Zhao Z, Hou YY, Ye XT, et al. Rottlerin exhibits anti-cancer effect through inactivation of S phase kinase-associated protein 2 in pancreatic cancer cells. Am J Cancer Res. 2016;6:2178–91. 27822410 PMC5088284

[59] Wang C, Zheng C, Wang H, Shui S, Jin H, Liu G, et al. Dual degradation mechanism of GPX4 degrader in induction of ferroptosis exerting anti-resistant tumor effect. Eur J Med Chem. 2023;247:115072. 36603510 10.1016/j.ejmech.2022.115072

[60] Jane EP, Premkumar DR, Pollack IF. Coadministration of sorafenib with rottlerin potently inhibits cell proliferation and migration in human malignant glioma cells. J Pharmacol Exp Ther. 2006;319:1070. 16959960 10.1124/jpet.106.108621

[61] Yuan S, Wei C, Liu G, Zhang L, Li J, Li L, et al. Sorafenib attenuates liver fibrosis by triggering hepatic stellate cell ferroptosis via HIF-1alpha/SLC7A11 pathway. Cell Prolif. 2022;55:e13158. 34811833 10.1111/cpr.13158 PMC8780895

[62] Huang F, Huang Z, Wei Q, Liu G, Pu J. E3 ubiquitin ligase HECTD3 is a tumor suppressor and mediates the polyubiquitination of SLC7A11 to promote ferroptosis in colon cancer. Exp Cell Res. 2023;430:113697. 37422058 10.1016/j.yexcr.2023.113697

[63] Chen Q, Zhang T, Zeng R, Zhang K, Li B, Zhu Z, et al. The E3 ligase TRIM7 suppresses the tumorigenesis of gastric cancer by targeting SLC7A11. Sci Rep. 2024;14:6655. 38509147 10.1038/s41598-024-56746-3 PMC10954695

[64] Wang Z, Shen N, Wang Z, Yu L, Yang S, Wang Y, et al. TRIM3 facilitates ferroptosis in non-small cell lung cancer through promoting SLC7A11/xCT K11-linked ubiquitination and degradation. Cell Death Differ. 2024;31:53–64. 37978273 10.1038/s41418-023-01239-5 PMC10781973

[65] Miao M, Pan M, Chen X, Shen J, Zhang L, Feng X, et al. IL-13 facilitates ferroptotic death in asthmatic epithelial cells via SOCS1-mediated ubiquitinated degradation of SLC7A11. Redox Biol. 2024;71:103100. 38484644 10.1016/j.redox.2024.103100 PMC10950698

[66] Sun X, Huang N, Li P, Dong X, Yang J, Zhang X, et al. TRIM21 ubiquitylates GPX4 and promotes ferroptosis to aggravate ischemia/reperfusion-induced acute kidney injury. Life Sci. 2023;321:121608. 36958437 10.1016/j.lfs.2023.121608 PMC11483487

[67] Zhang J, Qiu Q, Wang H, Chen C, Luo D. TRIM46 contributes to high glucose-induced ferroptosis and cell growth inhibition in human retinal capillary endothelial cells by facilitating GPX4 ubiquitination. Exp Cell Res. 2021;407:112800. 34487731 10.1016/j.yexcr.2021.112800

[68] Wang Z, Xia Y, Wang Y, Zhu R, Li H, Liu Y, et al. The E3 ligase TRIM26 suppresses ferroptosis through catalyzing K63-linked ubiquitination of GPX4 in glioma. Cell Death Dis. 2023;14:695. 37872147 10.1038/s41419-023-06222-z PMC10593845

[69] Zhang Y, Yang Y, Chen W, Mi C, Xu X, Shen Y, et al. BaP/BPDE suppressed endothelial cell angiogenesis to induce miscarriage by promoting MARCHF1/GPX4-mediated ferroptosis. Environ Int. 2023;180:108237. 37802009 10.1016/j.envint.2023.108237

[70] Li Y, Zhang F, Hu F, Tong R, Wen Y, Fu G, et al. ERK1-mediated GLYCTK2 phosphorylation promotes fructolysis to sustain glioblastoma survival under glucose deprivation. Cell Death Discov. 2025;11:266. 40467571 10.1038/s41420-025-02544-3 PMC12137673

