# Supplementary material for: Rottlerin triggers dual degradation of SLC7A11 and GPX4 to drive ferroptosis and chemosensitization in hepatocellular carcinoma
Source: Cell Death Discov. 2026 Jan 30;12:89. doi: 10.1038/s41420-026-02942-1 (PMC12877103; doi:10.1038/s41420-026-02942-1)

**Supplementary figures and figure legends for Luo et. al.**

**Supplementary Figure Legends**

**Supplementary Fig. 1 The** **Fe^2+^ and PUFAs levels between rottlerin-treated HCC cells and the control group. A** HLE treated with or without 5 μM rottlerin for 48 hours were stained with 5 µM PGSK probe for Fe^2+^ assays, followed by flow cytometry analysis. The representative images (left) and relative Fe^2+^ levels (right) were displayed. Data were represented as mean ± SD (n = 3) and analyzed using Student’s t test; ns means not significant. **B** HLE and LM3 cells were treated with 5 μM rottlerin for 0, 6, 12, and 24 hours. The PUFAs levels were determined by ELISA (enzyme-linked immunosorbent assay). Data were represented as mean ± SD (n = 3) and analyzed using One−way ANOVA; ns means not significant, **P* < 0.05.


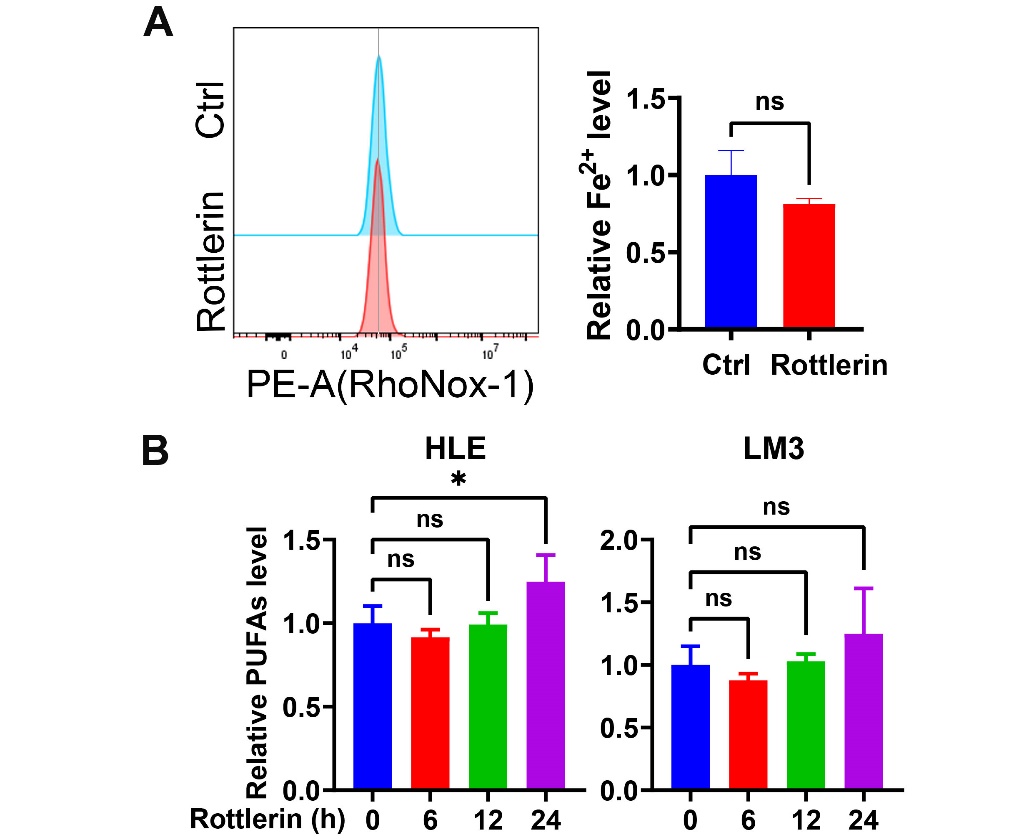


**Supplementary Fig. 1**

**Supplementary Fig. 2 Rottlerin induces HCC cell death not primarily dependent on PKCδ.** **A** PKCδ protein levels were detected by Western blot. **B** 5 μM rottlerin was added in Control (Ctrl) and PKCδ−knockout (KO#1 and KO#2) HLE cells for 48 hours, then cells were collected for PI staining and analyzed by flow cytometry. The representative images and cell death rates were displayed. The sgRNA sequences targeting PKCδ were listed below: KO #1: ATGAAGGAGGCGCTCAGCAC; KO #2: CAATGGCAAGGCTGAGTTCT. Data were represented as mean ± SD (n = 3) and analyzed using One−way ANOVA, ns means not significant, ****P* < 0.001.


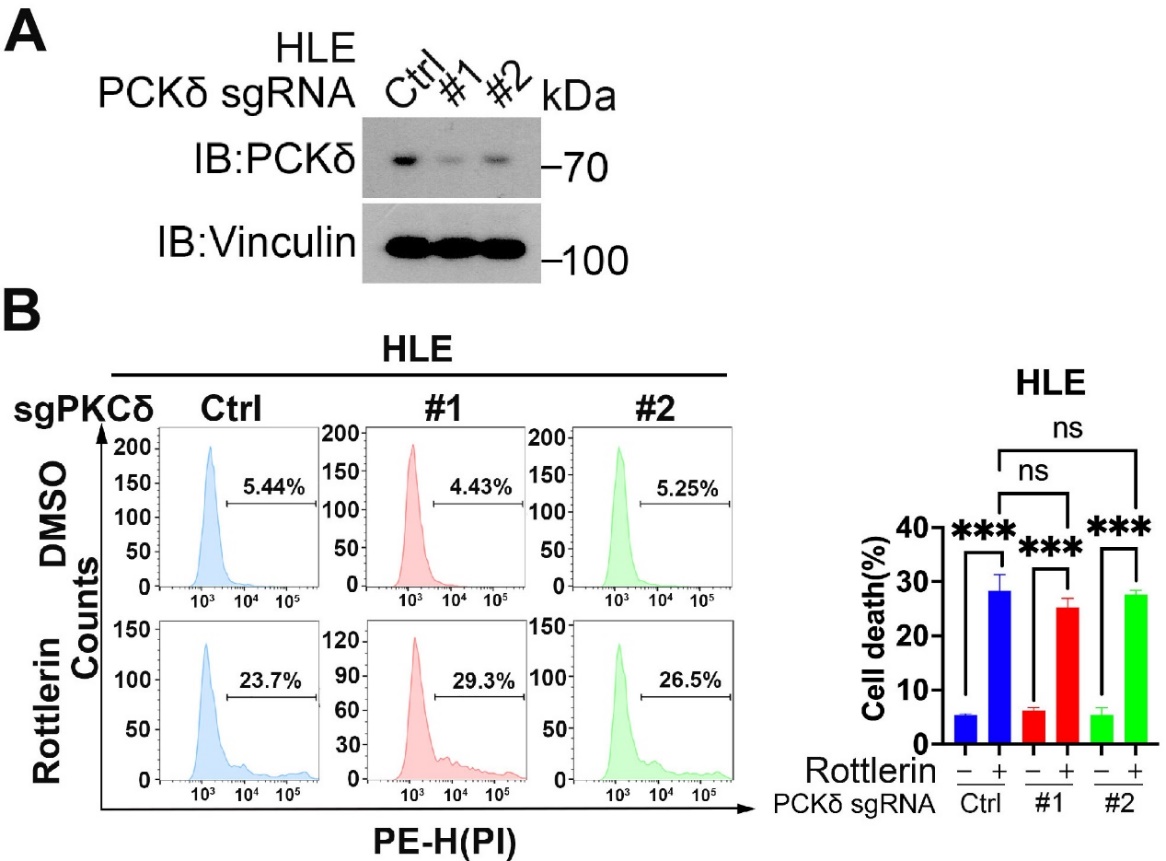


**Supplementary Fig. 2**

**Sample of FACS gating strategies for PI staining**

PI-positive cells are defined as cells with fluorescence greater than 99% of the unstained sample. An example of gating strategy for PI staining is provided as following:


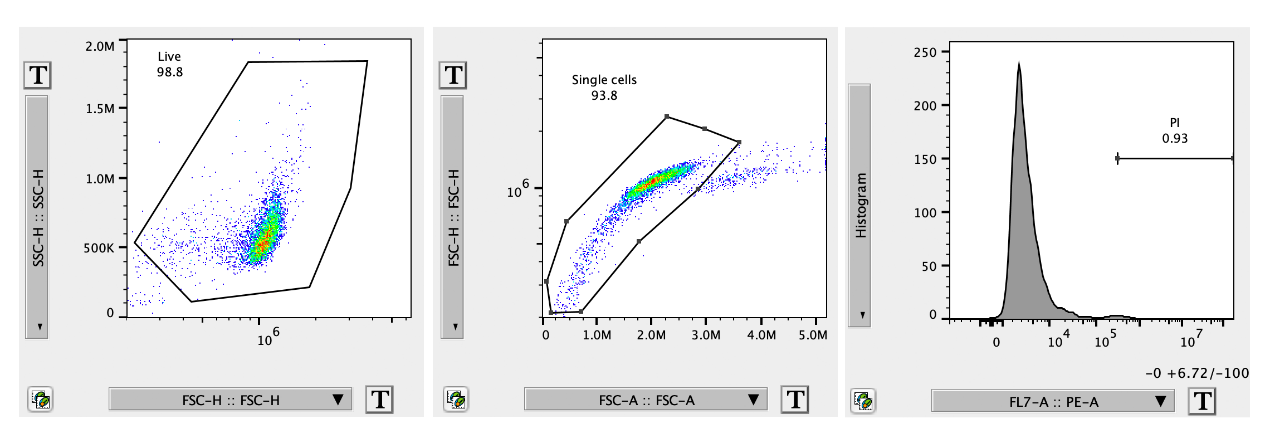

Supplement: Supplementary file 1 — Supplementary figures and figure legends [file 41420_2026_2942_MOESM1_ESM.docx]
