# Supplementary material for: Rottlerin triggers dual degradation of SLC7A11 and GPX4 to drive ferroptosis and chemosensitization in hepatocellular carcinoma
Source: Cell Death Discov. 2026 Jan 30;12:89. doi: 10.1038/s41420-026-02942-1 (PMC12877103; doi:10.1038/s41420-026-02942-1)

Figure 4A

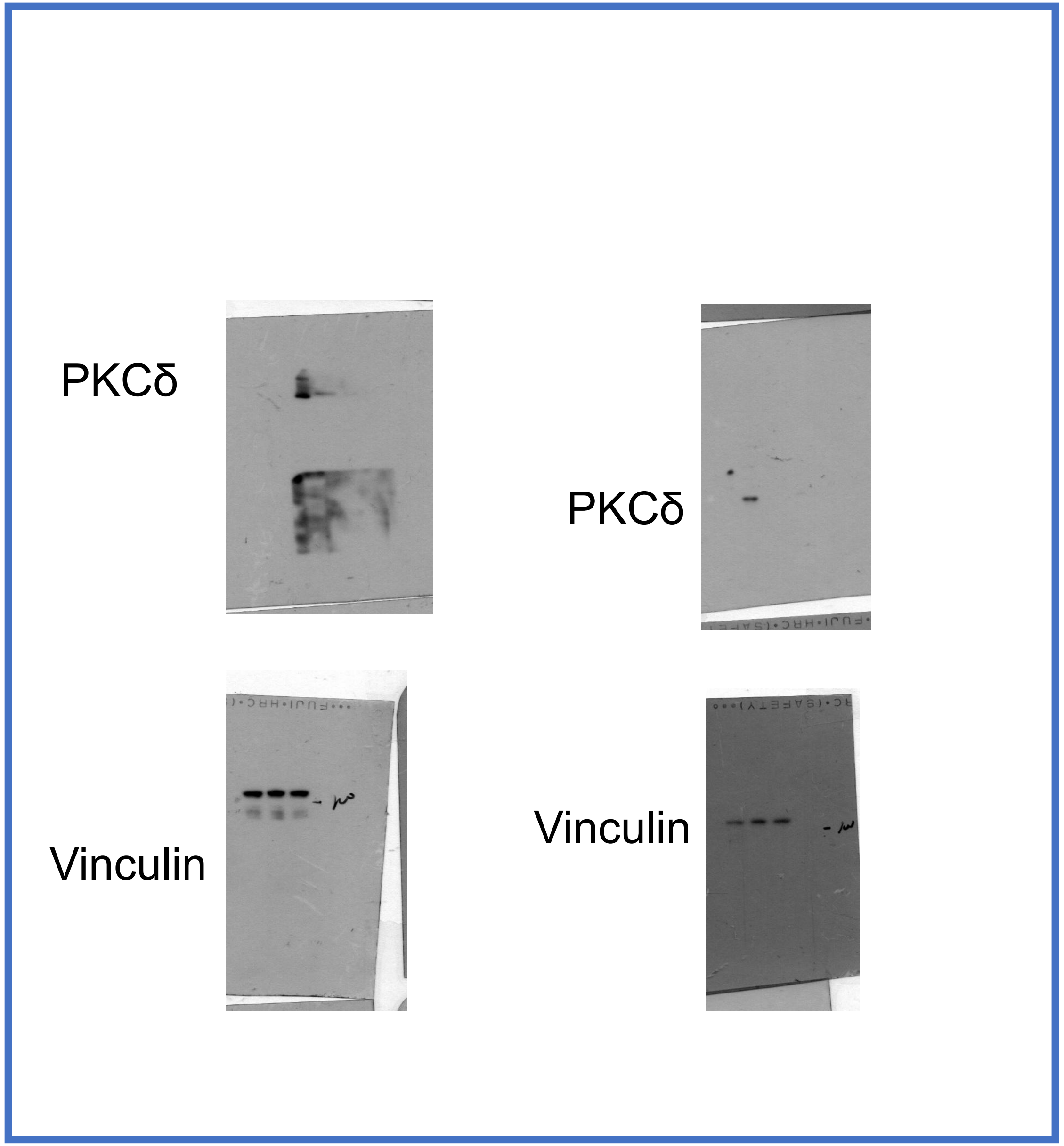

Figure 5

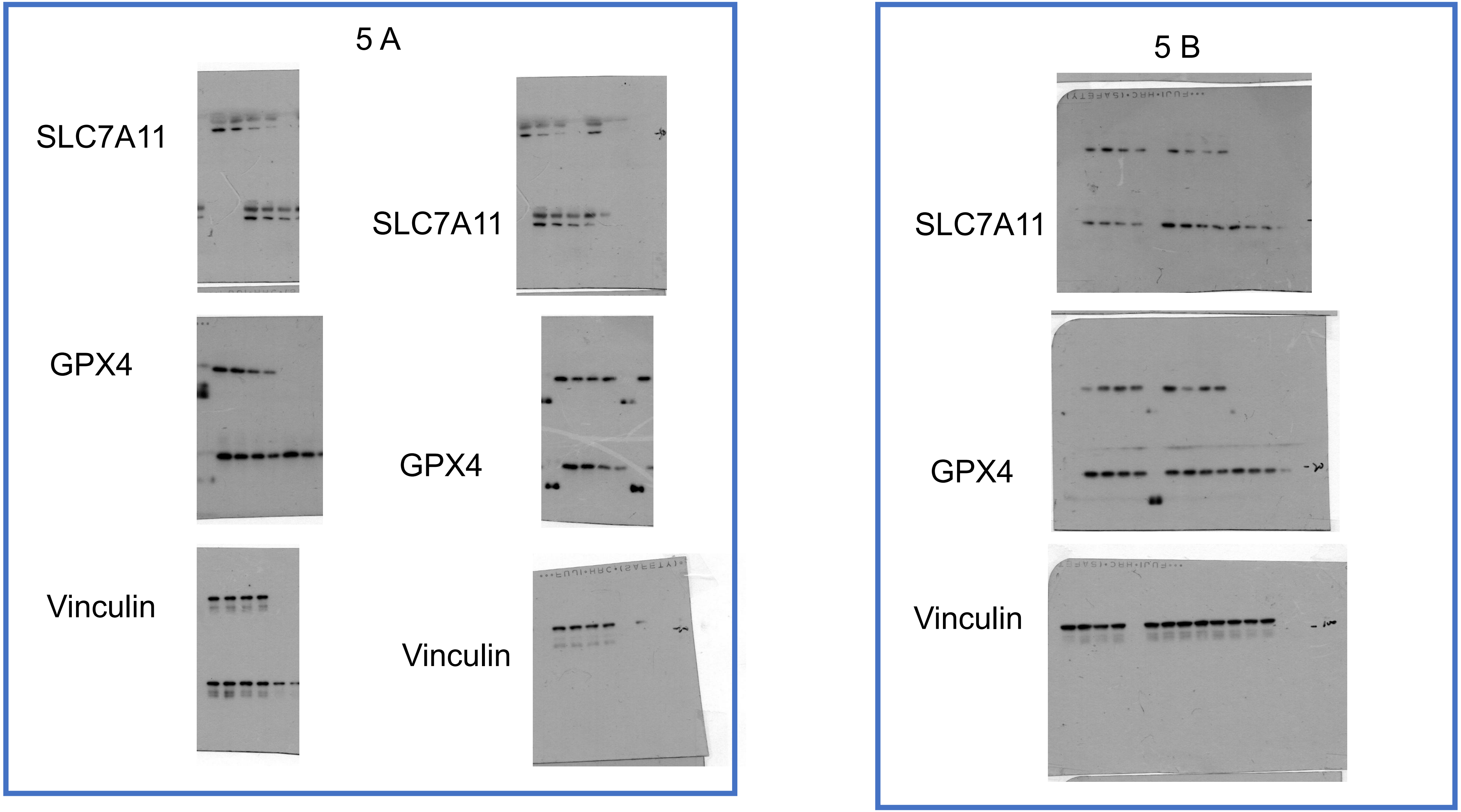
5 A
SLC7A11
SLC7A11
GPX4
GPX4
Vinculin
Vinculin
5 B
SLC7A11
GPX4
Vinculin

Figure 5

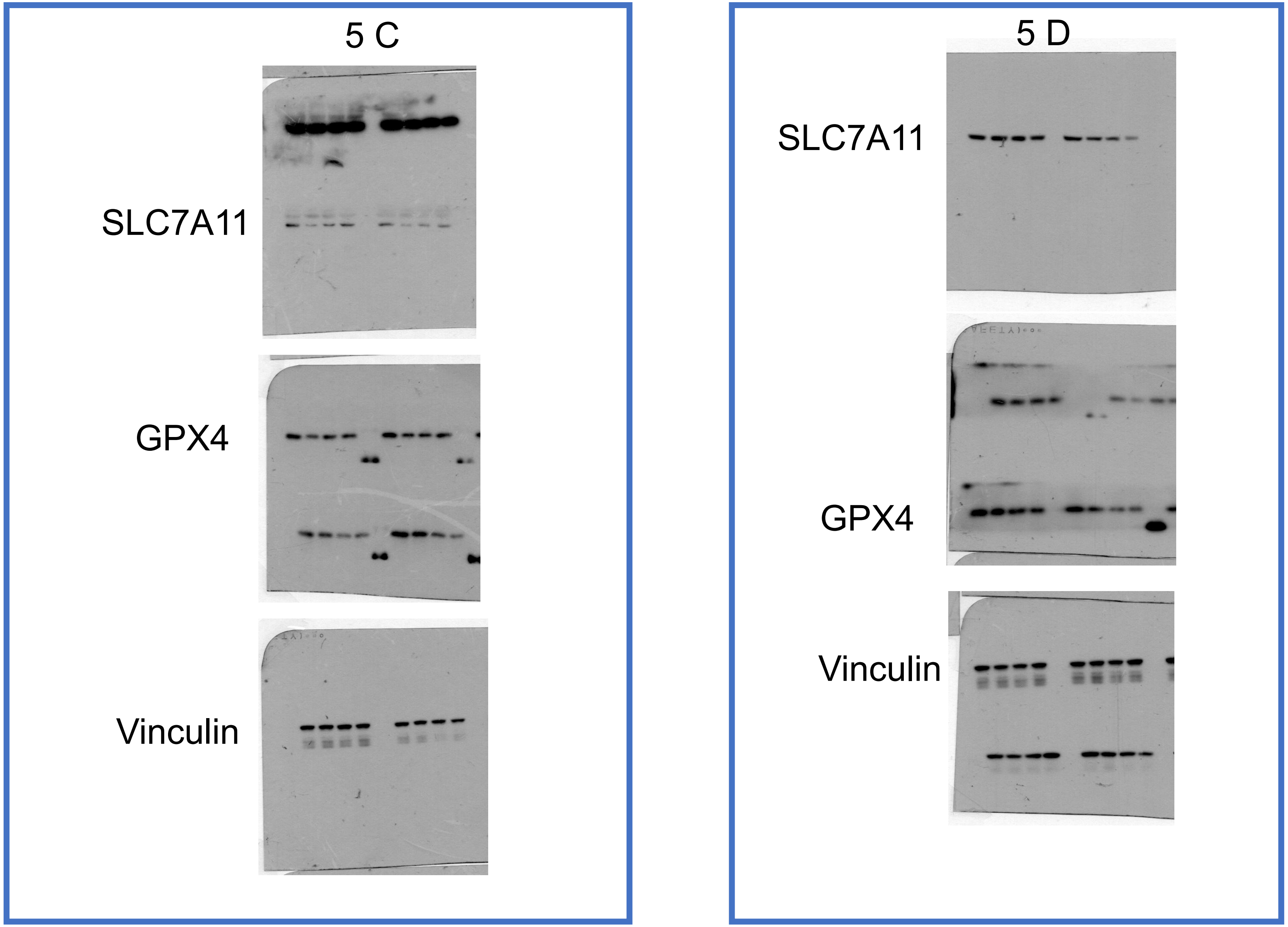

Figure 5F

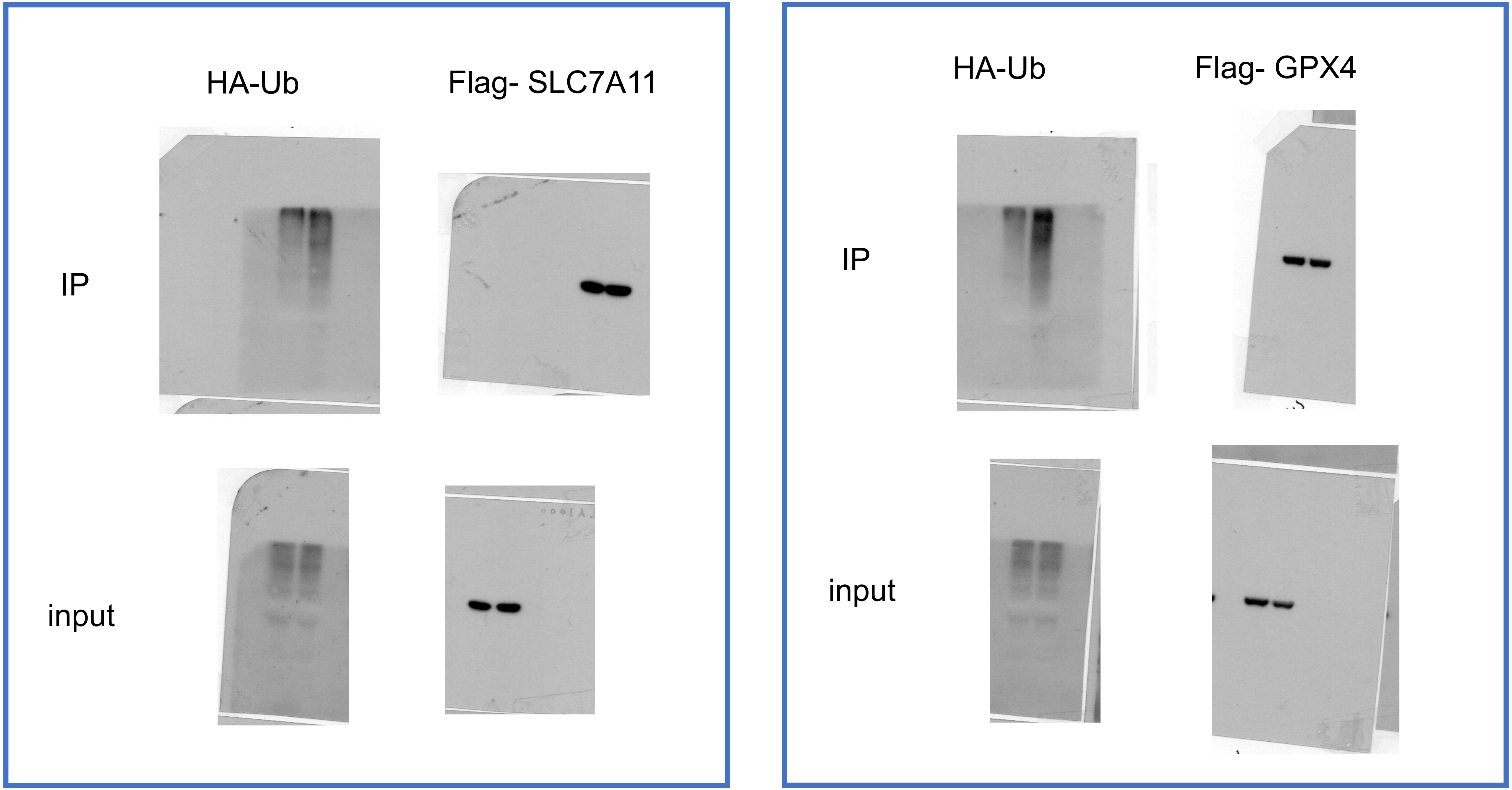

Figure 5G

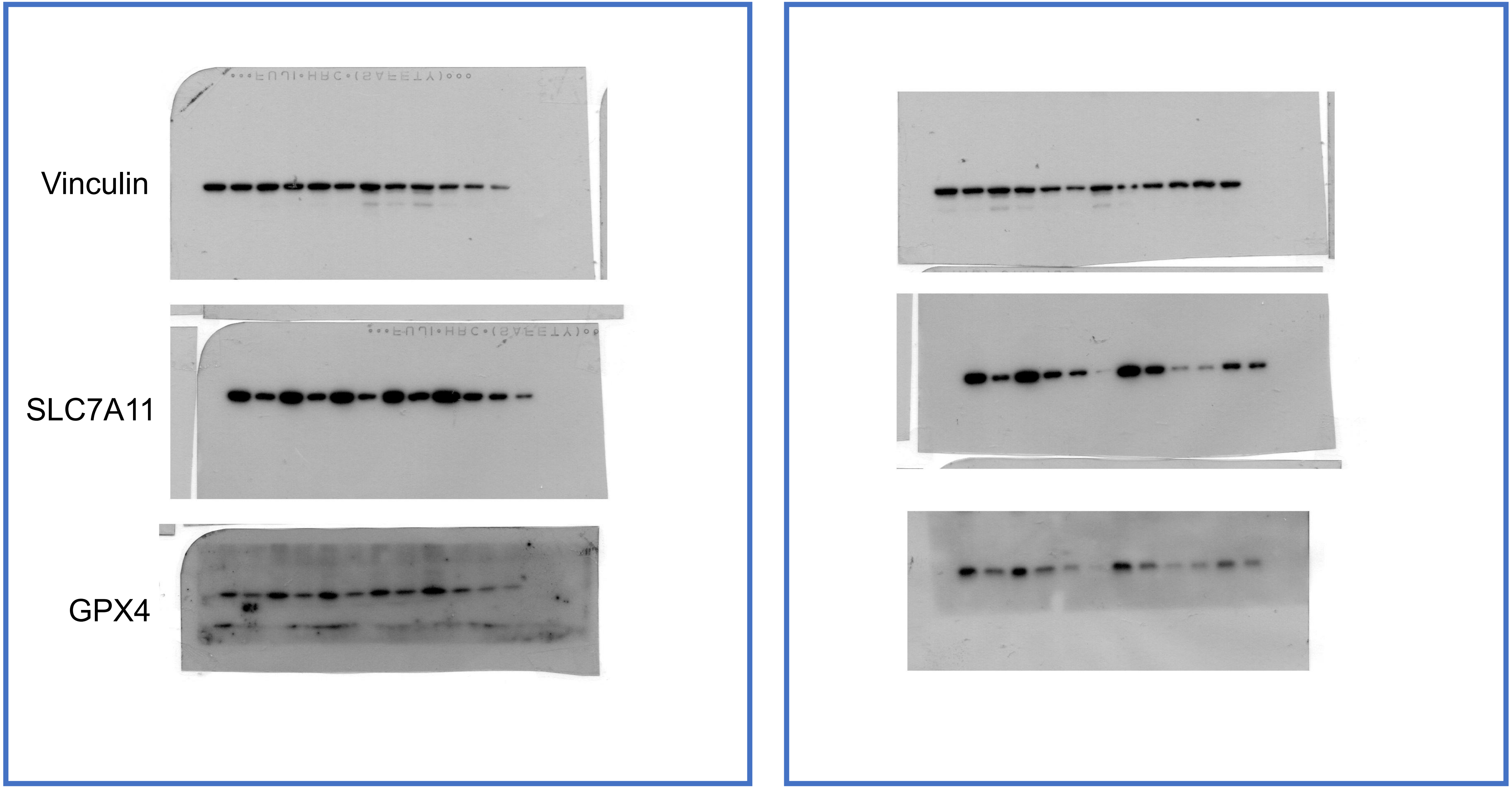

Figure 5H

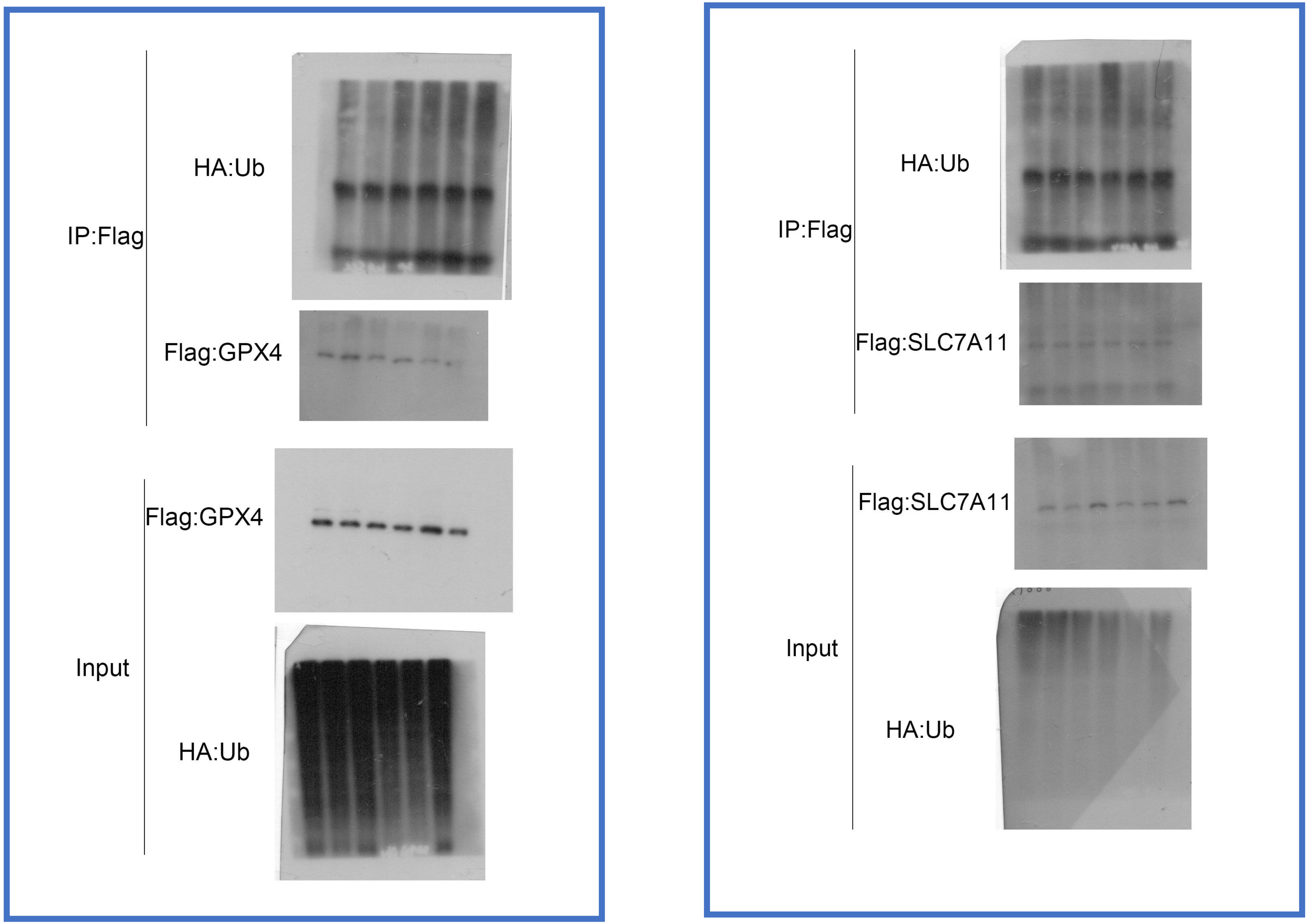

IP:Flag
HA:Ub
Flag:GPX4
Input
Flag:GPX4
HA:Ub
IP:Flag
HA:Ub
Flag:SLC7A11
Input
Flag:SLC7A11
HA:Ub

Figure 6C

SLC7A11

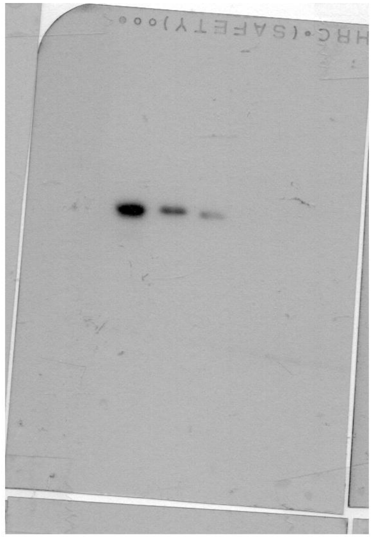

GPX4

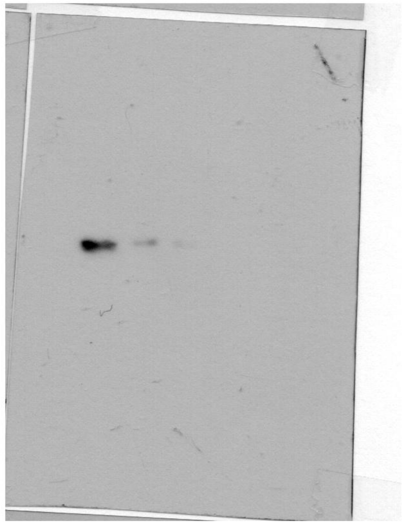

Vinculin

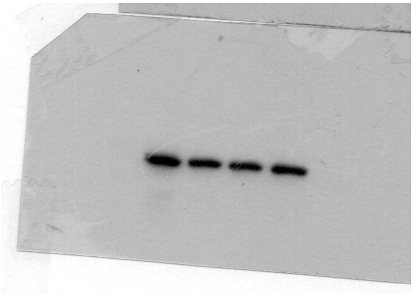

Supplementary Figure 2A

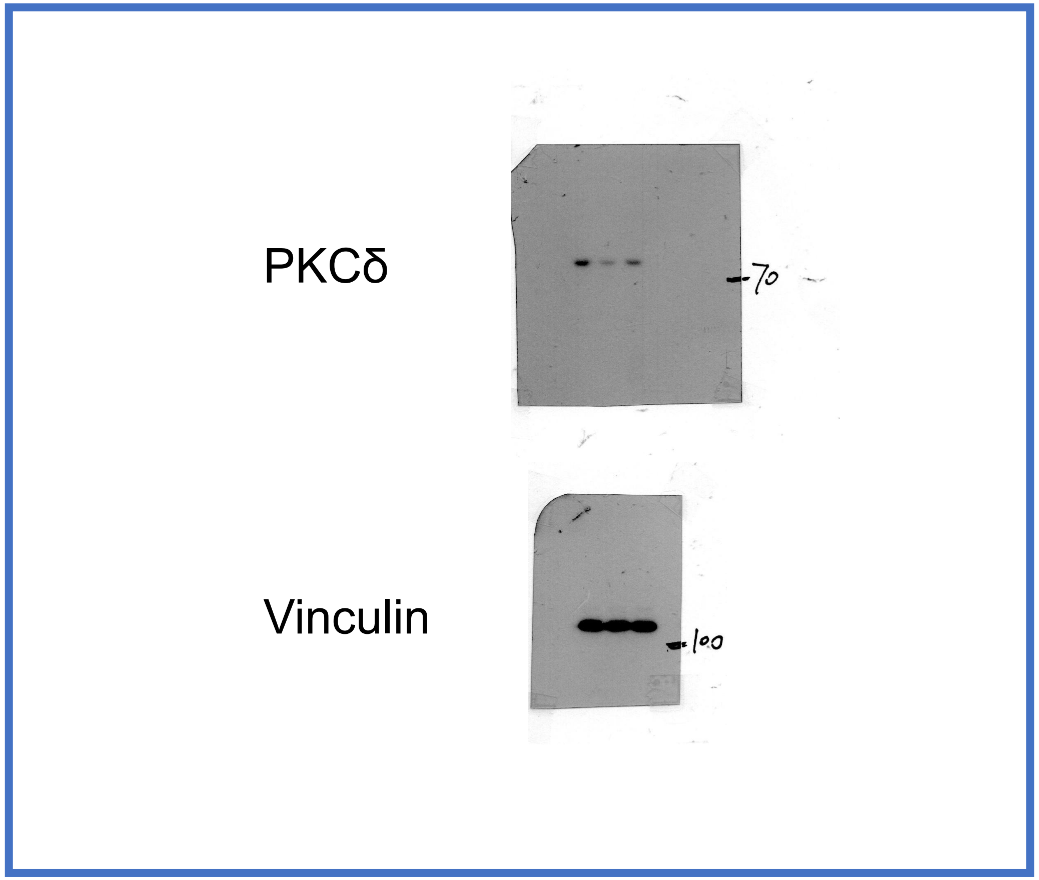

Supplement: Supplementary file 2 — uncropped original western blots [file 41420_2026_2942_MOESM2_ESM.pdf]
